# A Systems Genetics Approach Provides a Bridge from Discovered Genetic Variants to Biological Pathways in Rheumatoid Arthritis

**DOI:** 10.1371/journal.pone.0025389

**Published:** 2011-09-28

**Authors:** Hirofumi Nakaoka, Tailin Cui, Atsushi Tajima, Akira Oka, Shigeki Mitsunaga, Koichi Kashiwase, Yasuhiko Homma, Shinji Sato, Yasuo Suzuki, Hidetoshi Inoko, Ituro Inoue

**Affiliations:** 1 Division of Human Genetics, Department of Integrated Genetics, National Institute of Genetics, Mishima, Shizuoka, Japan; 2 Division of Molecular Life Science, School of Medicine, Tokai University, Isehara, Kanagawa, Japan; 3 Department of Human Genetics and Public Health, Institute of Health Biosciences, The University of Tokusima Graduate School, Tokushima, Tokushima, Japan; 4 Department of Laboratory, Japanese Red Cross Tokyo Blood Center, Koto-ku, Tokyo, Japan; 5 Department of Clinical Health Science, Tokai University School of Medicine, Isehara, Kanagawa, Japan; 6 Department of Internal Medicine, Division of Rheumatology, Tokai University School of Medicine, Isehara, Kanagawa, Japan; University of Oklahoma and Oklahoma Medical Research Foundation, United States of America

## Abstract

Genome-wide association studies (GWAS) have yielded novel genetic loci underlying common diseases. We propose a systems genetics approach to utilize these discoveries for better understanding of the genetic architecture of rheumatoid arthritis (RA). Current evidence of genetic associations with RA was sought through PubMed and the NHGRI GWAS catalog. The associations of 15 single nucleotide polymorphisms and *HLA-DRB1* alleles were confirmed in 1,287 cases and 1,500 controls of Japanese subjects. Among these, *HLA-DRB1* alleles and eight SNPs showed significant associations and all but one of the variants had the same direction of effect as identified in the previous studies, indicating that the genetic risk factors underlying RA are shared across populations. By receiver operating characteristic curve analysis, the area under the curve (AUC) for the genetic risk score based on the selected variants was 68.4%. For seropositive RA patients only, the AUC improved to 70.9%, indicating good but suboptimal predictive ability. A simulation study shows that more than 200 additional loci with similar effect size as recent GWAS findings or 20 rare variants with intermediate effects are needed to achieve AUC = 80.0%. We performed the random walk with restart (RWR) algorithm to prioritize genes for future mapping studies. The performance of the algorithm was confirmed by leave-one-out cross-validation. The RWR algorithm pointed to *ZAP70* in the first rank, in which mutation causes RA-like autoimmune arthritis in mice. By applying the hierarchical clustering method to a subnetwork comprising RA-associated genes and top-ranked genes by the RWR, we found three functional modules relevant to RA etiology: “leukocyte activation and differentiation”, “pattern-recognition receptor signaling pathway”, and “chemokines and their receptors”.

These results suggest that the systems genetics approach is useful to find directions of future mapping strategies to illuminate biological pathways.

## Introduction

Genome-wide association studies (GWAS) have identified a large number of novel genetic loci underlying susceptibility to common diseases [1], which leads to an interest in how these discoveries may be translated into improvement in health care and public health. Identification of associated variants can illuminate causal pathways and provide a clue for therapeutic targets [2]. Ultimately, it may be possible to predict the development of common diseases by genetic profiling, in which multiple genetic loci are simultaneously tested [3].

There are conflicting views regarding the usefulness of genetic variants in disease prediction [4]–[8]. The idea widely received is that the predictive ability of genetic profiling is limited with some exceptions [5] because most common genetic variants identified to date confer relatively small effects on disease risk and explain a small portion of the individual variation in disease risks [9]. The risk estimates will be updated and become more accurate with new genetic discoveries by conducting more large-scale GWAS [6] and by extending the analysis of low frequency and rare variants [10]. There are some examples that individually rare variants with relatively large effect contribute to complex trait variation [11]–[13]. It is important to infer the allelic architecture of as-yet-discovered risk variants on the basis of current evidence of known disease-associated variants in order to provide clues for future mapping strategies [14].

There are prerequisites for evidence-based genetic testing. First, a rigorous scientific basis for the genetic variants used for the genetic profiling is essential [15]. In fact, most of the genetic variants used by direct-to-consumer genetic testing to predict an individual's risk to common diseases have been shown to lack consistent evidence of gene-disease associations [15]. Second, and probably most importantly, the predictive ability of genetic variants should be evaluated [5]. The predictive ability can be quantified by several measures such as the area under the receiver operating characteristic curve [16]. Third, it is necessary to corroborate the generalizability of a genetic risk prediction model in independent datasets [17]. Systematic validation and characterization of the evidence of genetic associations at both discovery and translational phases of human genomics are also required [18], [19]. In these circumstances, meta-analysis can be a useful tool to improve the estimation of effect sizes of genetic variants by combining results from individual studies, thereby making it possible to evaluate variants for model inclusion in a rigorous way [20].

**Figure 1:**
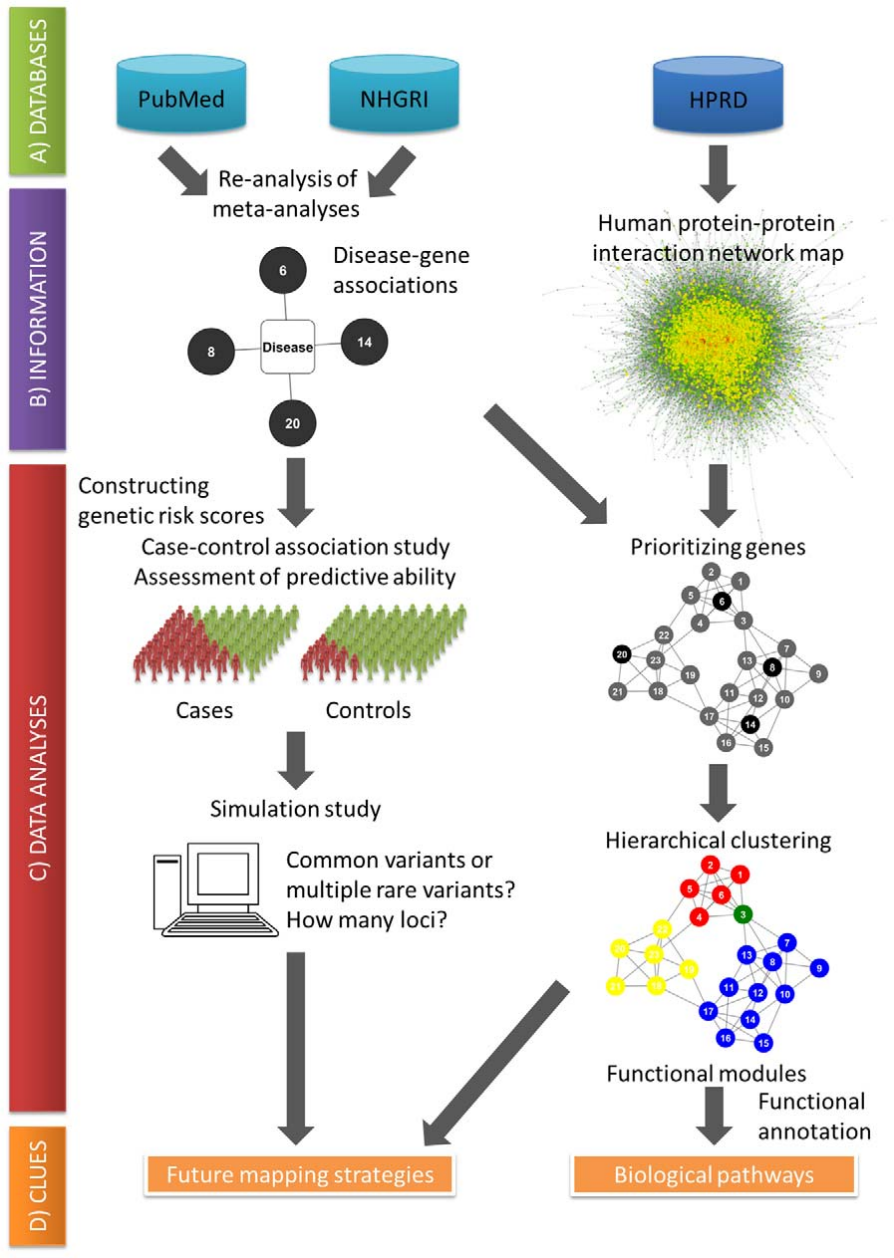
The systems genetics approach proposed in this study. A) Databases from which knowledge is extracted. Meta-analyses and GWAS findings are sought in PubMed and NHGRI GWAS catalog, respectively. Human protein-protein interaction data is obtained from HPRD. B) Retrieved information is used to create two types of networks: ‘gene-disease association network’ and ‘protein-protein interaction network’. C) The data analysis phase. The gene-disease associations are confirmed by using real case-control subjects. The predictive ability of selected genetic variants is evaluated and the result is used in the simulation study to infer allelic architecture of as-yet-discovered genetic variants. Two types of networks are integrated to prioritize genes by the global measure of distance to known disease-associated genes within the protein-protein interaction network. Hierarchical clustering algorithm is applied to a subnetwork comprising top-ranked genes and functional annotation for each cluster is used for the inference on biological pathways underlying the disease of interest. D) The systems genetics approach emerges two types of clues: Future mapping strategies, and biological pathways.

We propose here a systems genetics approach to utilize current evidence of genetic associations for better understanding of the genetic architecture of complex disease [21]. The outline of our approach is schematically shown in Figure 1 (The left and right columns correspond to the first three and last steps in the following description). First, genetic variants associated with the disease of interest are identified by exhaustively reviewing meta-analyses of genetic association studies. Second, the association and the predictive ability of the selected variants are confirmed in real case-control subjects. Third, a framework of simulation study is formulated to address how many additional loci should be mapped for the establishment of acceptable levels of genetic risk prediction. Fourth, a network analysis is implemented where information on disease-associated genes are integrated through human interactome such as the protein-protein interaction (PPI) network for the design of future mapping studies and exploring biological pathways [22].

We applied the systems genetics approach to rheumatoid arthritis (RA, [MIM 180300]). RA is a common autoimmune disease characterized by chronic, destructive and debilitating arthritis [23]. The etiology of RA is not completely known and most likely involves a complex interplay of both genetic and environmental factors. It has been shown that multiple alleles at the *HLA-DRB1* locus within the major histocompatibility complex (MHC) region are associated with RA. RA susceptibility loci outside the MHC region have been identified through candidate gene approaches and GWAS [24], [25]. The subdivision of RA patients in terms of the presence or absence of rheumatoid factor (RF) and antibodies against cyclic citrullinated peptide (anti-CCP) is increasingly recognized for possible prevention and treatment strategies. Genetic factors may also contribute to the phenotypic diversity in RA [26].

## Results

### Electronic database searches

We sought published meta-analyses that had evaluated the association between genetic variants and RA risk in population-based studies through two electronic databases: PubMed and NHGRI GWAS catalog. Figure S1A shows the outline of our literature search strategy using PubMed database. The reasoning for each of the excluded articles in the abstract reading, full-text search and data extraction stage is listed in Tables S1, S2, and S3, respectively. After selecting meta-analyses that fulfilled inclusion criteria, we found 29 articles addressing 27 variants located on 18 genetic loci [27]–[55]. After reducing redundant variants on the same genetic locus, 20 variants were identified (Text S1A). We also retrieved seven articles addressing the contribution of the *HLA-DRB1* locus [56]–[62].

**Figure 2:**
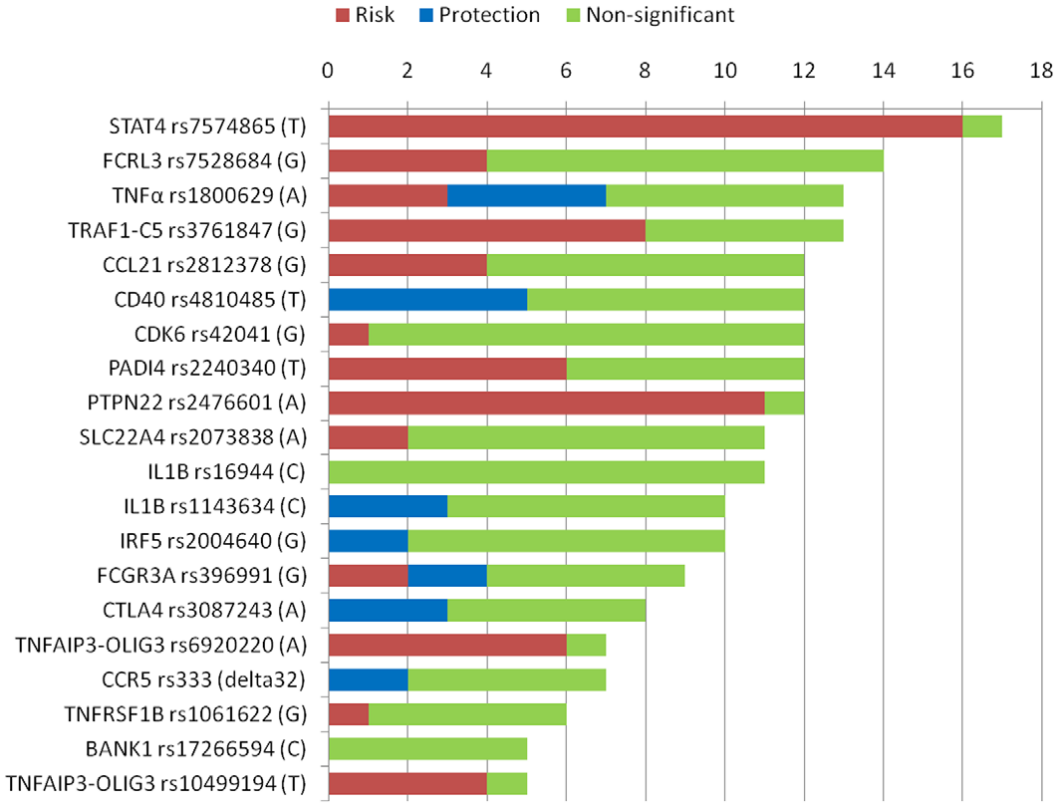
Overview of association studies in RA of 20 genetic variants examined in the meta-analyses met our inclusion criteria. Colored bars displays number of individual studies according to the result of testing for association of each variant with RA: red, studies show significant evidence of an increased risk; blue, studies show significant evidence of disease protection; and green, studies show non-significant result. The significance level was set at *P* = 0.05.

In order to overview the retrieved meta-analyses, we classified individual studies analyzing the same genetic variants into three groups: studies showing significant evidence of increased and reduced risk, or non-significant result (Figure 2). In cases of single nucleotide polymorphism (SNP) rs7574865 at the *STAT4* locus, rs2476601 at the *PTPN22* locus, and rs6920220 and rs10499194 at the *TNFAIP3-OLIG3* locus, consistent lines of evidence of associations were observed. In the other cases, more than half of the individual studies did not show significant evidence of association. However, the direction of associations in the studies showing significant evidence was consistent for each variant except for rs1800629 at *TNF-α* and rs396991 at *FCGR3A*. This result suggests that most of the individual studies may be underpowered to detect small genetic effect [63], [64]. Thus, conclusions derived from meta-analyses may be useful to select genetic variants for risk prediction models.

The outline of the NHGRI GWAS catalog search is shown in Figure S1B. Eight articles were retrieved [52], [65]–[71]. We found the 61 associations with *P*<1.0×10^−5^: 7 for the HLA region and 54 for the non-HLA region comprising 34 distinct genetic loci. Restricting the statistical significance level at *P*<5.0×10^−8^, 18 associations, 10 of which did not overlap those from the PubMed search, were retrieved. All of the retrieved associations were derived from meta-analyses of several GWAS and replication studies [69]–[71].

### Re-analysis of published meta-analyses and selection of genetic variants

We re-analyzed the meta-analyses addressing 20 genetic associations (Table S4; Text S1A). For each meta-analysis, a median of 6,758 cases (interquartile range [IQR]: 3,445–10,994) and 7,643 (IQR: 3,367–14,406) controls had been involved. We found that there were 10 meta-analyses showing statistically significant between-study heterogeneity. This indicates that the between-study heterogeneity was more frequent than what would be expected by chance (*P* = 7.2×10^−6^). The median of *I* ^2^ metric was 40.4% (IQR: 16.1–63.9%). In 14 of 20 meta-analyses, the genetic associations passed the significance threshold of *P* = 2.5×10^−3^ under the fixed effects model meta-analysis in the overall populations. Even when applying the random effects model meta-analysis, which is a conservative approach under the presence of between-study heterogeneity, evidence of association was confirmed in all of the 14 polymorphisms (*P*<0.05).

From the PubMed search, we identified the following 14 variants that fulfilled our selection criteria: rs7574865 (*STAT4*); rs3087243 (*CTLA4*); rs7528684 (*FCRL3*); rs3761847 (*TRAF1-C5*); rs2812378 (*CCL21*); rs4810485 (*CD40*); rs42041 (*CDK6*); rs2240340 (*PADI4*); rs2476601 (*PTPN22*); rs2073838 (*SLC22A4*); rs2004640 (*IRF5*); rs6920220 and rs10499194 (*TNFAIP3-OLIG3*); and rs333 (*CCR5*). Among these 14 polymorphisms, 13 were SNPs and one was the 32 bp-deletion polymorphism in *CCR5* (referred to as rs333).

For the *HLA-DRB1* alleles, we selected six alleles that were significantly associated with RA risk in a comprehensive review article [62] using the largest collection of relevant articles: HLA-DRB1*01:01, DRB1*09:01, DRB1*10:01, DRB1*04:04, DRB1*04:01, and DRB1*04:05.

We identified an additional 10 SNPs from the NHGRI GWAS catalog: rs3093024 (*CCR6*); rs874040 (*RBPJ*); rs11676922 (*AFF3*); rs13017599 (*REL*); rs6859219 (*ANKRD55*); rs934734 (*SPRED2*); rs2736340 (*BLK*); rs26232 (*C5orf30*); rs13315591 (*FAM107A*); and rs706778 (*IL2RA*).

Collectively, 23 SNPs, one deletion polymorphism, and six *HLA-DRB1* alleles that were significantly associated with RA risk were identified. Allele frequencies of the genetic variants with validated associations with RA were highly differentiated between East Asian and European populations (Table S5). Among them, 15 SNPs with minor allele frequency greater than 5% in Japanese and six *HLA-DRB1* alleles were selected through our database searches.

### Ethnic differences

We examined the ethnicity-specific effects of these variants (Table S6). We found heterogeneity in the odds ratios (ORs) between ethnic groups at *P*<0.05 for rs2240340 (*PADI4*) and rs7528684 (*FCRL3*). The OR of rs2240340 was larger for East Asian (OR = 1.31, 95% confidence interval [CI]; 1.22–1.41, *P* = 5.6×10^−13^) than for European descent populations (OR = 1.03, 95% CI; 0.99–1.07, *P* = 0.16). Similarly, the rs7528684 association was stronger for East Asian populations (OR = 1.16, 95% CI; 1.09–1.24, *P* = 7.8×10^−6^) than for European descent populations (OR = 1.03, 95% CI; 0.98–1.09, *P* = 0.27). The effects observed with East Asian populations were used in the genetic risk score (Table 1).

**Table 1:** Association analysis of RA with selected genetic variants.

Gene	SNP	A1/A2^A^	Univariate^B^		Multivariate^B^		Previous report^C^
			OR (95% CI)	*P*	OR (95% CI)	*P*	OR (95% CI)
*HLA-DRB1*	*01:01	+/−	1.29 (1.03–1.61)	0.025	1.95 (1.52–2.48)	8.8×10^−8^	1.60 (1.39–1.84)
	*09:01	+/−	1.20 (1.04–1.39)	0.012	1.74 (1.48–2.04)	1.8×10^−11^	1.67 (1.44–1.94)
	*10:01	+/−	2.88 (1.42–5.83)	3.3×10^−3^	3.59 (1.72–7.52)	7.0×10^−4^	2.35 (1.90–2.91)
	*04:01	+/−	1.89 (1.23–2.90)	3.9×10^−3^	2.70 (1.69–4.30)	3.0×10^−5^	3.30 (3.01–3.61)
	*04:04	+/−	1.49 (0.55–4.02)	0.43	2.92 (0.98–8.67)	0.054	1.85 (1.54–2.22)
	*04:05	+/−	2.31 (2.01–2.66)	1.3×10^−31^	2.80 (2.40–3.27)	9.4×10^−39^	3.84 (3.30–4.46)
**SNPs with strong evidence of association (** ***P*** **<2.5×10^−3^)**							
*CCR6*	rs3093024	A/G	1.25 (1.12–1.39)	4.1×10^−5^	1.26 (1.13–1.42)	6.3×10^−5^	1.19 (1.15–1.24)
*PADI4*	rs2240340	T/C	1.23 (1.11–1.37)	1.5×10^−4^	1.24 (1.11–1.40)	2.6×10^−4^	1.31 (1.22–1.41)
*BLK*	rs2736340	T/C	1.24 (1.10–1.39)	3.2×10^−4^	1.24 (1.09–1.41)	7.8×10^−4^	1.19 (1.13–1.27)
*CD40*	rs4810485	T/G	0.80 (0.72–0.89)	4.7×10^−4^	0.82 (0.73–0.92)	7.8×10^−4^	0.87 (0.83–0.90)
**SNPs with nominally significant association signals (** ***P*** **<0.05)**							
*C5orf30*	rs26232	T/C	0.86 (0.77–0.98)	0.018	0.86 (0.75–0.98)	0.021	0.90 (0.87–0.94)
*SLC22A4*	rs2073838	A/G	1.14 (1.02–1.27)	0.022	1.17 (1.04–1.32)	0.012	1.11 (1.05–1.18)
*AFF3*	rs11676922	T/A	1.11 (1.00–1.24)	0.043	1.11 (0.99–1.24)	0.083	1.14 (1.10–1.18)
*FCRL3*	rs7528684	G/A	1.11 (1.00–1.24)	0.047	1.08 (0.96–1.21)	0.20	1.16 (1.09–1.24)
**SNPs showing the same direction of effect**							
*SPRED2*	rs934734	G/A	1.14 (0.99–1.31)	0.064	1.17 (1.00–1.36)	0.043	1.13 (1.09–1.17)
*STAT4*	rs7574865	T/G	1.10 (0.98–1.23)	0.093	1.09 (0.97–1.23)	0.14	1.23 (1.19–1.27)
*CTLA4*	rs3087243	A/G	0.92 (0.82–1.04)	0.18	0.96 (0.84–1.10)	0.56	0.89 (0.85–0.95)
*TRAF1*	rs3761847	A/G	1.05 (0.95–1.17)	0.35	1.03 (0.91–1.15)	0.66	1.13 (1.09–1.17)
*IL2RA*	rs706778	T/C	1.05 (0.95–1.17)	0.36	1.05 (0.93–1.17)	0.43	1.12 (1.09–1.16)
**SNPs showing the opposite direction of effect**							
*TNFAIP3*	rs10499194	T/C	1.18 (0.96–1.46)	0.11	1.18 (0.94–1.48)	0.15	0.82 (0.77–0.87)

A A1 and A2 represent the coded and non-coded alleles, respectively.

B ORs and 95% CIs were estimated by logistic regression analyses using univariate analysis for each allele and then using multivariate analysis including all the alleles. The number of coded alleles (A1) was used as the predictor value in the logistic regression analyses.

C ORs and 95% CIs were calculated by meta-analyses of published studies: *HLA-DRB1* from [62]; *CD40*, *SLC22A4*, *STAT4*, *CTLA4*, *TRAF1*, *TNFAIP3*, and *IRF5* from re-analysis of meta-analyses shown in Table S4; *PADI4* and *FCRL3* from re-analysis of ethnicity-specific meta-analyses shown in Table S6; and *CCR6*, *BLK*, *C5orf30*, *AFF3*, *SPRED2*, and *IL2RA* from original GWASs [69]–[71]. These ORs were used to create genetic risk scores.

### Association analysis

We conducted a case-control study of 1,287 RA cases and 1,500 controls in Japanese (see Materials and Methods for description of our cohort). Genotype counts for six *HLA-DRB1* alleles and 15 SNPs are shown in Table S7. For the SNPs, the missing genotype rates were small (at most 1.3% for rs2736340). SNP rs2004640 (*IRF5*), which deviated from the HWE in controls at *P*<0.001, was excluded from subsequent analyses.

We assessed the association of each genetic variant with RA risk by logistic regression analysis (Table 1). For the *HLA-DRB1* alleles, HLA-DRB1*04:05 allele showed highly significant evidence of association with the risk of RA. It should be noted that the ORs of all the *HLA-DRB1* alleles in the multivariate logistic regression analysis were larger than those in the univariate analysis. When an allele was evaluated in the univariate analysis, the other five putative risk alleles were grouped together into one referent group, which resulted in a weakened association signal.

For the SNPs, strong evidence of association was observed with rs3093024 in *CCR6* (*P* = 4.1×10^−5^, OR = 1.25), rs2240340 in *PADI4* (*P* = 1.5×10^−4^, OR = 1.23), rs2736340 in *BLK* (*P* = 3.2×10^−4^, OR = 1.24), and rs4810485 in *CD40* (*P* = 4.7×10^−4^, OR = 0.80). We found that four SNPs showed nominally significant associations at *P*<0.05 for rs26232 (*C5orf30*), rs2073838 (*SLC22A4*), rs11676922 (*AFF3*), and rs7528684 (*FCRL3*). SNPs on *SPRED2* and *STAT4* showed suggestive associations at *P*<0.1. SNPs on *CTLA4*, *TRAF1-C5*, and *IL2RA* had the same direction of effect as identified in previous studies. SNP rs10499194 on *TNFAIP3-OLIG3* showed the opposite direction of effect. The observed opposite direction of rs10499194 seems to be attributable to the difference in linkage disequilibrium between marker and true disease allele across populations. Shimane et al. showed a similar result and identified a non-synonymous SNP (rs2230926) in *TNFAIP3* associated with RA [72].

The ORs for SNPs obtained with the univariate analysis were similar to those with the multivariate analysis, indicating that the associations of these SNPs are independent association signals. These results suggest that a substantial proportion of the loci identified in the meta-analyses are likely to be shared across populations.

### Discrimination using genetic risk models

This study is reported in accordance with the Strengthening the Reporting of Genetic Risk Prediction Studies recommendations [73]. With the use of the receiver operating characteristic (ROC) curve, we calculated the area under the ROC curve (AUC) to evaluate the predictive ability of the genetic risk scores based on the selected variants (see Materials and Method for description of the construction of genetic risk score). The AUC for the HLA model was 65.9% (95% CI, 63.9 to 67.9%). The non-HLA model including 14 SNPs showed an AUC of 58.8% (56.6 to 60.9%). The AUC for the integrative model was 68.4% (66.4 to 70.4%). The addition of 14 SNPs to the *HLA-DRB1* alleles increased the AUC by 2.5%. The observed increase in the AUC was statistically significant (*P* = 2.8×10^−6^). The integrative model shows better fit than the HLA model in terms of Akaike's information criterion (Table 2). We examined an *ad hoc* model, where rs10499194 on *TNFAIP3-OLIG3* showing the opposite effect as identified in previous studies was removed. The AUC was then 68.6 (66.6 to 70.6%). The improvement in the AUC from the integrative model was statistically significant (*P* = 0.034).

**Table 2:** The discriminative ability and the global model fit of three predictive models according to subphenotype of case patients.

Case phenotype	Model	AUC (95% CI)	AIC^A^
Overall	HLA	65.9 (63.9–67.9)	3477.7
	Non-HLA	58.8 (56.6–60.9)	3630.7
	Integrative	68.4 (66.4–70.4)	3421.9
RF & anti-CCP positive	HLA	68.3 (65.2–71.4)	1603.7
	Non-HLA	60.0 (56.7–63.3)	1694.2
	Integrative	70.9 (67.8–73.9)	1578.0

A Akaike's information criterion.

We performed the same ROC analyses by using only the patients with both anti-CCP and RF positivity (Table 2). The AUC for the HLA, non-HLA and integrative models was 68.3% (65.2 to 71.4%), 60.0% (56.7 to 63.3%), and 70.9% (67.8 to 73.9%), respectively. For each genetic risk model, the AUC in both RF and anti-CCP positive patients versus controls was greater than that in overall patients versus controls. The result of the association study for anti-CCP and RF positive RA is shown in Table S8.

**Figure 3:**
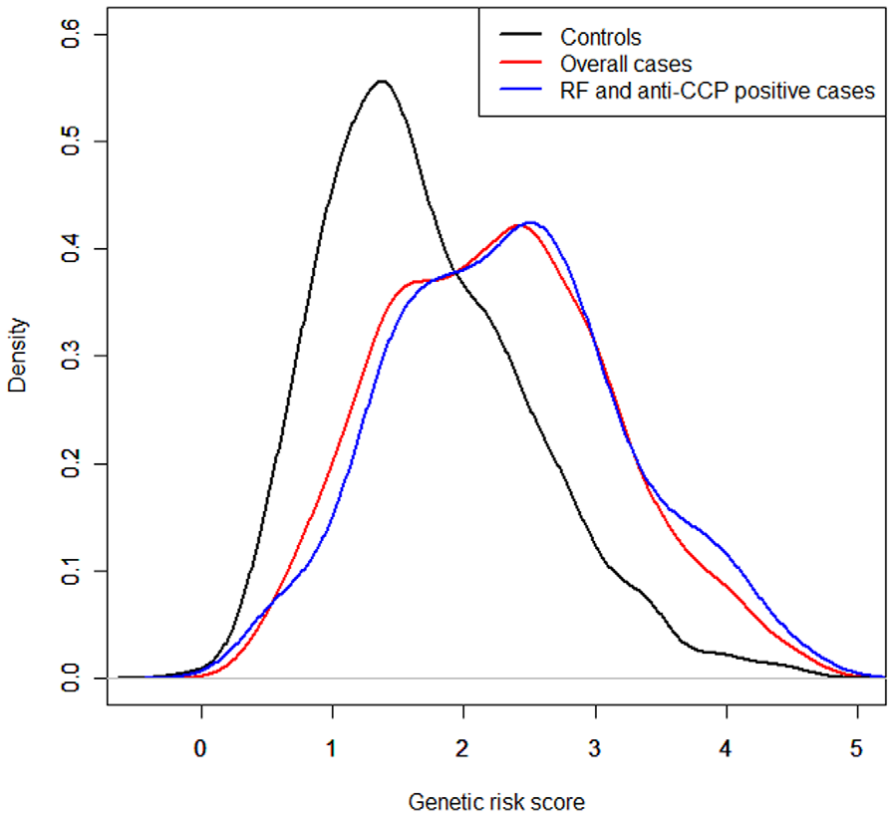
Distribution of risk scores by phenotypic status for the integrative model, in which six *HLA-DRB1* alleles and 14 SNPs were included. The curves were generated with a Gaussian kernel density smoother.

Figure 3 depicts the distribution of genetic risk scores by phenotypic status for the integrative model. The distribution of the genetic risk scores in cases differs from that in controls (*P* = 1.1×10^−61^). The curve in RF and anti-CCP positive cases shifts upward compared to the curve in overall cases, indicating that the risk scores in RF and anti-CCP positive cases were larger than those in overall cases. This was reflected in better discrimination ability between both RF and anti-CCP positive patients and controls (AUC = 70.9%) than that between overall cases and controls (AUC = 68.4%). Each curve in Figure 3 looks like multimodal distribution. The multimodality of these curves is attributable to differences in genetic risk score among the *HLA-DRB1* genotypes.

### Simulation study: How many additional loci should be mapped?

We investigated how many additional loci are required to achieve an acceptable level of genetic risk prediction via simulation study. We set AUC of 80.0% as an acceptable level based on the diagnostic accuracy of anti-CCP antibody and RF for RA. According to a recent meta-analysis [74], the pooled sensitivity and specificity were 67% and 95% for anti-CCP antibody, respectively, and 69% and 85% for IgM RF, respectively. The naïve estimate of the AUC was 81% for anti-CCP antibody and 75% for IgM RF.

We simulated the distribution of RA risks in the general population based on observed ORs and allele frequencies for the selected variants (see Materials and Methods, and Text S1B for details). For the base model in which 13,392,312 multi-locus genotypes generated by combining the six *HLA-DRB1* alleles and the 14 SNPs are included, the simulated AUC was 71.0%. The AUC of the base model was similar to that observed in anti-CCP and RF positive patients (AUC = 70.9%). Starting with the base model, we evaluated the simulated AUC value assuming that hypothetical additional loci were discovered.

**Figure 4:**
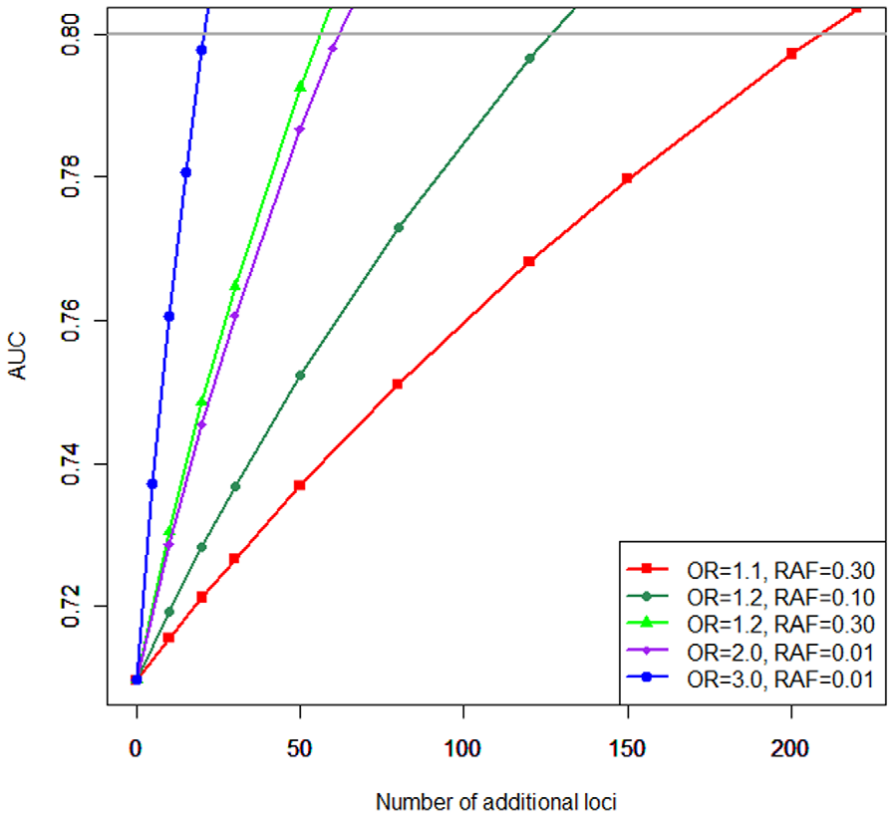
Simulation study addressing how many additional loci should be mapped for the establishment of excellent genetic risk prediction. Five scenarios with different combination of OR and RAF were examined.

Result of the simulation study is shown in Figure 4. Under the common disease-common variant hypothesis, ∼50 loci are needed in the setting of additional loci with OR = 1.2 and risk allele frequency (RAF) of 0.30. Taking into consideration the fact that the ORs from recent GWAS of RA were close to 1.1, a scenario of OR = 1.1 and RAF = 0.30 may be more realistic. In this scenario, ∼220 loci are required. When assuming the multiple rare variants with intermediate effects that remain undiscovered and setting the additional loci with OR of 3.0 and RAF of 0.01, only ∼20 loci are sufficient for AUC of 0.80. When assuming OR = 2.0 and RAF = 0.01, an additional 50 loci are needed.

We further implemented simulations in which combination of common and rare variants was examined. When assuming *HLA-DRB1* alleles, 150 loci with OR = 1.1 and RAF = 0.30, and 10 loci with OR = 3.0 and RAF = 0.01, the AUC was 80.2%. The simulation rendered AUC = 95.2% under the assumption of *HLA-DRB1* alleles, 300 loci with OR = 1.1 and RAF = 0.30, and 140 loci with OR = 3.0 and RAF = 0.01.

### Network analysis

The simulation study shows that many additional variants need to be discovered. We hypothesized that variants within genes on the same biological pathways of known RA susceptibility genes can be associated with RA. Then, we performed following network analyses to prioritize genes for future mapping studies.

We constructed the PPI network by using HPRD database [75], [76]. The PPI network contained 37,080 interactions between 9,521 human proteins. The selected variants were assigned to a single protein-coding gene (Table S5; Text S1C). There are 19 RA-associated proteins mapped in the PPI network (HLA-DRB1, STAT4, FCRL3, TRAF1, CCL21, CD40, CDK6, PTPN22, SLC22A4, IRF5, CTLA4, TNFAIP3, CCR6, REL, SPRED2, BLK, FAM107A, and IL2RA).

**Figure 5:**
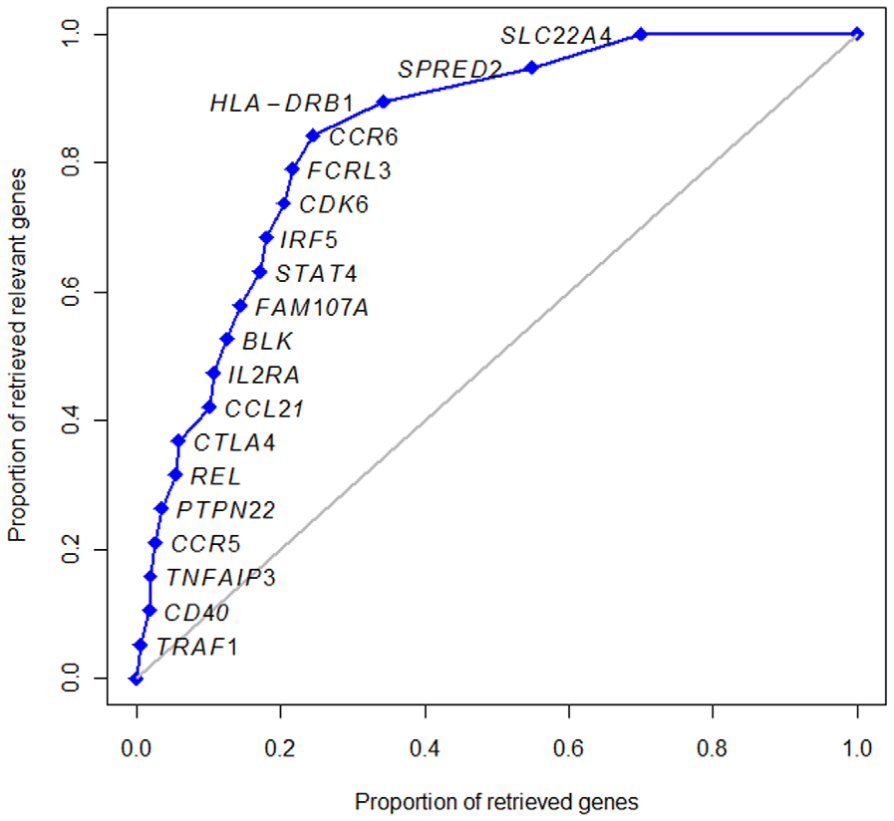
ROC curve using the leave-one-out cross-validation method to evaluate the predictive ability of the RWR algorithm. The gray diagonal line corresponds to the AUC of 0.5 and no discrimination (i.e., random performance).

We used the random walk with restart (RWR) algorithm [77] to prioritize genes in terms of the proximity to the validated RA susceptibility genes within the PPI network (see Materials and Method for details). As a preliminary test, we confirmed that the value of restart probability, *r*, did not largely affect the ranking of genes. When we examined different values of *r* (0.3, 0.5, and 0.7), the spearman's rank correlation coefficients ranged from 0.967 to 0.993. The predictive ability of the network-guided gene prioritization method was evaluated by the leave-one-out cross-validation. As shown in Figure 5, most of the left-out genes are highly evaluated. For example, *TRAF1* ranked 58th among 9,503 genes evaluated. The AUC by the leave-one-out cross-validation was 84.4%, indicating an excellent predictive ability. This result also suggests that the RA-associated genes are in proximity to each other within the PPI network.

In the top-ranked genes, we can find many genes that may be involved in the susceptibility to RA and other autoimmune diseases. The RWR algorithm points to *ZAP70* in the first rank. Notably, a mutation in *ZAP70* is identified to cause chronic autoimmune arthritis in mice [78]. Sakaguchi et al. [78] demonstrate that the mutation in the mouse *ZAP70* affects thymic T-cell selection and leads to the development of RA-like arthritis. ZAP70 has direct interactions with PTPN22 and FCRL3 among proteins encoded by RA-associated genes in the HPRD database. FCRL3 has a direct interaction only with ZAP70 in the HPRD database, which might cause upward bias in the ranking of *ZAP70*. Even when excluding *FCRL3* from the list of seed vertices, *ZAP70* ranked 42th among 9,503 genes, indicating that the priority of the gene is robust and that ZAP70 is located proximal to proteins encoded by the RA-associated genes in the PPI network. We found that *CD247*, *IL2RB* and *IL2* ranked 32th, 34th and 39th, respectively, and have been associated with RA in follow-up study of GWAS [79], [80] and studies exploring shared susceptibility loci among autoimmune diseases [81], [82]. *CD80*, *FCGR2A*, *FCGR2B*, *ICAM1*, *JAK2*, *LYN*, *NFKBIA*, *PTPN11*, *STAT3* and *TRAF3IP2* were shown to be associated with other autoimmune diseases according to the NHGRI GWAS catalog and systematic review [83].

**Figure 6:**
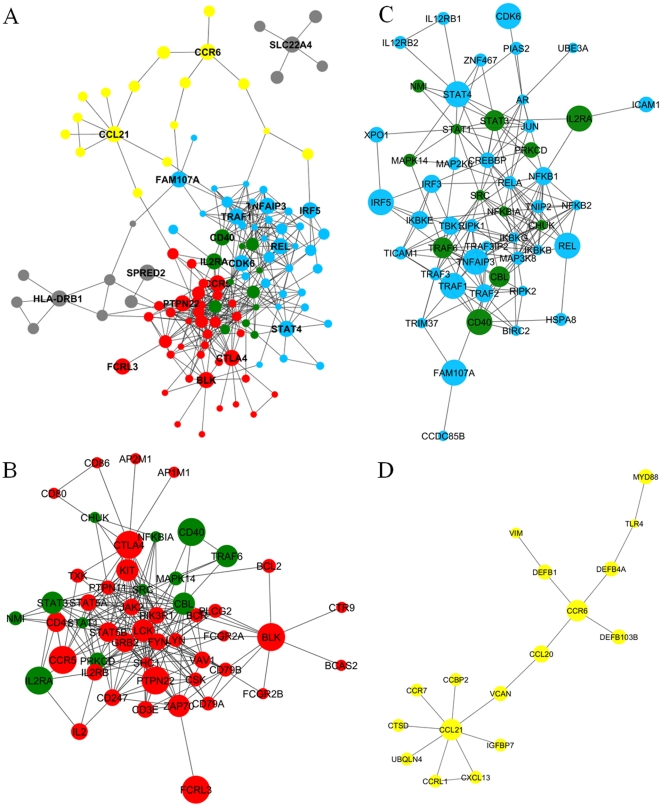
RA-associated network. A) Entire RA-associated network comprising known RA-associated genes and genes ranked in the top 100 by the RWR algorithm and edges are physical interactions between their products. Nodes are color coded by hierarchical clusters detected by the EAGLE algorithm: CL1, red; CL2; cyan, and CL3, yellow. Overlapped region between CL1 and CL2 are rendered in green. Node size is based on the ranking in the RWR algorithm. Official gene symbols are shown for known RA-associated genes. B–D) Subnetworks corresponds to the hierarchical clusters CL1-3.

Figure 6A depicts an RA-associated network that is a subnetwork of the PPI network in which vertices are the RA-associated genes and genes ranked in the top 100 by the RWR algorithm and edges are physical interactions between their products. In order to detect functional modules in the RA-associated network, we applied the EAGLE algorithm [84] and found three complexes each containing more than 10 vertices (referred to as CL1-3). The CL1 and CL2 overlap each other.

We further explored functional annotations of these three clusters by using DAVID [85], [86] (Table 3). The three clusters fitted into different categories of immunological pathway. CL1 can be assigned to an immunological pathway “leukocyte activation and differentiation” according to Gene Ontology (GO) terms annotated to genes in CL1 (Figure 6B). CL2 is associated with “pattern-recognition receptor signaling pathways” since genes in CL2 are enriched for GO terms and KEGG pathways such as Toll-like receptor and Nod-like receptor signaling pathways (Figure 6C). CL3 is enriched for genes relevant to “chemokines and their receptors” (Figure 6D). This result shows that the exploration of topology of the network based on curated disease susceptibility genes is useful to find functional modules involved in disease pathology. We confirmed that similar biological pathways are observed when the number of top ranked genes included into the RA-associated network is altered to 50 and 150 (Figures S2, S3, Tables S9, S10).

**Table 3:** Top-ranked GO and KEGG annotations for three clusters in RA-associated network.

Annotation	Term^A^	Count^B^	%^C^	FE^D^	P-value
**Cluster 1**					
GO:0045321	Leukocyte activation	20	40.0	23.3	1.4×10^−21^
GO:0002521	Leukocyte differentiation	15	30.0	32.3	8.0×10^−18^
hsa04660	T cell receptor signaling pathway	15	30.0	15.7	1.1×10^−13^
GO:0006468	Protein amino acid phosphorylation	19	38.0	8.0	2.8×10^−12^
**Cluster 2**					
hsa04620	Toll-like receptor signaling pathway	20	40.0	18.2	2.0×10^−20^
hsa04622	RIG-I-like receptor signaling pathway	14	28.0	21.7	7.5×10^−15^
GO:0007249	I-kappaB kinase/NF-kappaB cascade	12	24.0	31.6	4.3×10^−14^
hsa05200	Pathways in cancer	20	40.0	5.4	2.0×10^−10^
hsa04623	Cytosolic DNA-sensing pathway	10	20.0	22.0	2.0×10^−10^
hsa04621	NOD-like receptor signaling pathway	11	22.0	15.1	8.7×10^−10^
**Cluster 3**					
GO:0006935	Chemotaxis	9	52.9	47.6	1.9×10^−12^
GO:0007626	Locomotory behavior	9	52.9	27.8	1.5×10^−10^
GO:0006955	Immune response	11	64.7	13.5	2.7×10^−10^
GO:0006952	Defense response	10	58.8	13.7	3.1×10^−9^
GO:0019957	C-C chemokine binding	4	23.5	231.8	4.4×10^−7^
GO:0016493	C-C chemokine receptor activity	4	23.5	231.8	4.4×10^−7^

A Within each cluster, related terms are not shown to reduce redundancy. Among terms with parent-child relationships, we selected one showing highest significance enrichment P-value.

B Number of GO or KEGG category genes in each cluster.

C Percentage of GO or KEGG category genes in each cluster.

D Fold Enrichment of genes in each cluster compared to a background list.

## Discussion

The phenomenon named ‘missing heritability’ has received much attention [9] and calls into substantive question the usefulness of genetic profiles for disease risk prediction. In this study, we performed a systematic approach to overview and validate current evidence of genetic associations with RA and utilized them to find directions of future mapping strategies.

One fundamental question is whether genetic risk factors for RA overlap across ethnic groups [87]. Although the associations of two SNPs (*PADI4* and *FCRL3*) were significantly stronger in East Asian than in European, these two SNPs represented significant association in the overall populations and the recent European GWA meta-analysis [70] captured weak association signals of these SNPs. This suggests that these SNPs may be common risk factors but that their contribution to RA risk may differ across ethnic groups. Furthermore, we confirmed that most of the selected genetic variants from meta-analyses and NHGRI GWAS catalog were consistently replicated in a case-control study of 1,287 RA cases and 1,500 controls of Japanese. These results suggest that a substantial proportion of the loci identified in the meta-analyses are likely to be shared across populations.

The predictive ability of genetic variants for the development of RA was moderate: the AUC for the integrative model was 68.4% (66.4 to 70.4%). Notably, the AUC improved to 70.9% (67.8 to 73.9%) when we used patients with both RF and anti-CCP positivity. This finding is consistent with European study (AUC = 71% [68 to 73%] for anti-CCP positive RA) although the list of selected variants used was slightly different [88]. This is the first study showing that the predictive ability of genetic variants for RA mainly derived from European GWAS is similar between European and non-European populations by using a substantial number of case and control subjects. However, the predictive ability is suboptimal at the current stage.

When we implemented a simulation study addressing how many additional loci should be mapped, we set a goal of genetic risk prediction that achieves a similar level of accuracy with anti-CCP antibody for RA. Such genetic risk prediction may have clinical utility: when patients have primary symptoms such as joint pain and stiffness, prior knowledge of their higher genetic risks for RA may inspire them to undergo highly specific diagnostic tests such as anti-CCP antibody. Early detection and treatment can prevent severe disability for many patients.

According to the simulation study, more than 200 loci with OR = 1.1 and RAF = 0.30 are required to achieve AUC of 80.0%, implying that efforts relying only on GWAS may be reaching limits for improving predictive ability. With the advent of the development of massively parallel DNA sequencing technologies, exploring rare variants of large effect has attracted increased attention [89]. There is some evidence of rare variants with a large impact on risk of RA and autoimmune diseases [13], [90], [91]. Functionally defective rare variants in *SIAE* were recently shown to be associated with autoimmune diseases including RA with ORs estimated at approximately 8.0 [91]. In our simulation study, additional 20 rare but not private variants (minor allele frequency of 1%) with intermediate effect (OR of 3.0) suffice for AUC of 80.0%. Several hundreds of cases and controls must be resequenced for the discovery of such rare variants. However, whole-genome and whole-exome sequencing of large samples are costly and otherwise infeasible. The use of bar-coded multiplexed and target enrichment sequencing of the exonic regions of hundreds of candidate genes [92], [93] could be an alternative strategy if appropriate candidate genes were selected.

We applied the RWR algorithm to prioritize genes by using information on the list of curated RA-associated genes and the PPI network from HPRD database. The predictive ability of the RWR algorithm was proved to be excellent based on the leave-one-out cross-validation by omitting each RA-associated gene (AUC = 84.4%). This result suggests that the RA-associated genes are in proximity to each other within the PPI network, which is consistent with the recent study showing that the products of RA-associated genes are more interconnected than would be expected by chance [94]. The top-ranked genes with the RWR algorithm are intriguing. The gene in the first position (*ZAP70*) is a causal gene of RA-like autoimmune arthritis in mice [78]. Furthermore, recessive and compound heterozygous mutations in *ZAP70* cause human severe combined immunodeficiency [95]–[97]. Within the top 100 ranked genes, genes implicating susceptibility to RA (*CD247*, *IL2RB* and *IL2*) and to other autoimmune diseases (*CD80*, *FCGR2A*, *FCGR2B*, *ICAM1*, *JAK2*, *LYN*, *NFKBIA*, *PTPN11*, *STAT3* and *TRAF3IP2*) are enriched.

We also found that the analysis of network topology was useful to find functional modules involved in the disease pathology. This method has two steps: first, a disease-related network comprising genes in the vicinity to curated susceptibility genes within the PPI network is constructed using the RWR algorithm. Second, the overlapping and hierarchical structure of the disease-related network is explored and functional annotation is implemented for each cluster. The systems genetics approach proposed here will be applicable to most common diseases and will work well especially when genes associated with the disease of interest are in proximity to each other within the PPI network. When applying the method to RA, the resulting clusters were fitted into different categories of immunological pathways.

CL1 is related to “leukocyte activation and differentiation” (Figure 6B, Table 3). T-cell differentiation plays an important role in autoimmunity. Strongly self-reactive T cells are primarily eliminated in the thymus by negative selection (central tolerance). Some of the self-reactive T cells, however, may escape from negative selection and can cause autoimmune diseases. Defect in thymic T-cell selection due to a mutation of *Zap70* causes autoimmune arthritis in mice [78]. CL2 fits into “pattern-recognition receptor signaling pathways” (Figure 6C, Table 3). The innate immune functions of macrophages and neutrophils depend on pattern-recognition receptors such as Toll-like receptors and Nod-like receptors. Genes relevant to these pattern-recognition receptor signaling pathways were enriched in CL2. CL3 corresponds to “chemokines and their receptors” (Figure 6D, Table 3). The main function shared by chemokines and chemokine receptors is leukocyte chemotaxis, which helps direct migration of leukocytes to an injury site. Genetic defects in these biological pathways can inappropriately activate immune cells leading to inflammation and host cell destruction that can cause autoimmune diseases. Notably, the clusters inferred from this study are similar to the pathways implicated by Zhernakova et al. [83], in which genes associated with autoimmune diseases are grouped into four categories: ‘T cell differentiation’, ‘immune-cell activation and signaling’, ‘innate immunity and TNF signaling’, and ‘cytokines and chemokines’.

Some limitations of our study should be noted. Our electronic database search had been performed a year ago (on June 18 2010). Continuing efforts to examine whether more updated genetic findings appear in publication to renew the list of susceptibility genes to RA is required. By searching for the NHGRI database deposited after June 18 2010, we found four relevant articles.[80], [98]–[100] Genetic variants outside the MHC region passing the genome-wide significant threshold (5.0×10^−8^) were retrieved. Two Asian GWASs detected SNPs on two genetic loci (*AIRE*, and *PADI4*) [98], [99]. European GWAS identified 8 loci as shared genetic factors between RA and celiac disease [80]. We reconsidered our network analysis by including newly discovered loci. We could assign these 10 loci to 8 unique genes: *AIRE*, *PADI4*, *TRAF1*, *STAT4*, *YDJC*, *UBASH3A*, *CD247*, and *ATXN2*. *TRAF1*, *STAT4*, and *PADI4* were already included into our model. *YDJC* is not deposited in the HPRD database. Thus, 4 genes (*AIRE*, *UBASH3A*, *CD247*, and *ATXN2*) were newly included. We re-examined the RWR algorithm using a total of 23 genes as initial vertices. The rank correlation in genes between before and after including the 4 genes was 0.978. This indicates the ranking of candidate genes were not largely affected. The result from the hierarchical clustering method and functional annotation rendered similar biological pathways (Figure S4, Table S11). As previously mentioned, *CD247* was one of top-ranked genes (32th) in the original RWR analysis. The result of network analysis by using additional 4 newly discovered genes converged on the same biological pathways, suggesting the strong relevance of these pathways to the etiology of RA. Only physical PPI data were used to construct the molecular network and it is inevitably noisy and incomplete. A PPI network integrated with a transcription profiling network could improve the predictive ability of network-guided prioritization of genes [22], [101].

We have demonstrated that recent successful discoveries of genetic variants associated with diseases are valuable resources to provide targets for future resequencing studies to reveal the biological pathways. Such efforts utilizing GWAS discoveries will accelerate genetic discoveries and improve the predictive ability of the genetic variations. While exploration of other types of genomic variation such as rare and low frequency single nucleotide changes and insertions and deletions of nucleotides is promising, it may be challenging because the variants are likely to be population-specific. The biological pathways highlighted by the various common genetic variants associated with the disease across populations will encourage examination and functional annotation of newly discovered rare variants.

## Materials and Methods

### Ethics statement

The Ethics Committee of Tokai University approved the study protocols and all participants gave written informed consent.

### Study participants

1,287 RA subjects and 1,500 control subjects of Japanese origin were recruited. All cases were diagnosed by board certified rheumatologists and fulfilled 1987 American College of Rheumatology criteria [102]. The dataset was updated from our previous study [103].

Information on the positivity of anti-CCP and RF for 481 and 462 cases, respectively, was measured. Anti-CCP antibody titers were measured with the second generation ELISA kit (MESACUP CCP; Medical & Biological Laboratories Co. Ltd, Nagoya, Japan). A cut-off value of 4.5 U/ml was used for anti-CCP antibody positivity. RF positivity was determined by using N-Assay TIA RF Nittobo (Nitto Boseki Co., Ltd, Koriyama, Japan). The positivity of anti-CCP and RF was observed in 90.4% and 80.5% cases, respectively.

### Genotyping

All study participants were genotyped for *HLA-DRB1* alleles and selected SNPs described below. Genotyping of *HLA-DRB1* alleles was performed by Luminex Multi-Analyte Profiling system (xMAP) with a WAKFlow HLA typing kit (Wakunaga, Hiroshima, Japan). Genotyping of SNPs was performed by TaqMan SNP Genotyping Assays on the ABI PRISM 7900HT Sequence Detection System (Applied Biosystems, Tokyo, Japan). Departure from Hardy-Weinberg equilibrium (HWE) in control samples was examined at the significance level of *P*<0.001 by means of the exact test using PLINK software [104].

### Electronic database search strategies

#### PubMed search

We identified published meta-analyses addressing the association between genetic variants and RA risk in population-based studies. We performed a literature search of the PubMed database (last search June 18, 2010). Searches were conducted using the following keywords: “rheumatoid arthritis” and [genetic(s) or polymorphism(s) or allele(s) or mutation(s)] and (meta-analysis or metaanalysis or “systematic review”). Reference mining of retrieved articles was used to identify additional articles. Meta-analyses included in our analysis had to meet all of the following criteria: evaluated RA risk as the outcome (analyses of pharmacogenomics and RA severity were excluded) and published in English. Two researchers (HN and TC) conducted literature searches independently, and any disagreement between the two researchers was accommodated by the third researcher (AT).

Genetic models and methods for combining studies examined in the retrieved meta-analyses differed according to article. In order to evaluate evidence of association of genetic variant with RA in the same statistical manner, we performed re-analysis of published meta-analyses. Therefore, we included the following meta-analyses: greater than or equal to five independent studies were included and the total number of cases and controls was larger than 3,000; adequate data to calculate OR for each of the included studies was provided; and per-allele effect of risk allele was examined. When there were several meta-analyses on the same variant including different studies, we created a comprehensive set of individual studies using following criteria: i) studies did not overlap data between studies, and ii) study with largest sample size was used when studies overlapped data.

#### The NHGRI GWAS catalog

Recent update of GWAS findings was sought by the NHGRI GWAS catalog (http://www.genome.gov/gwastudies/ Accessed June 18, 2010). We included the associations that met the genome-wide significance of *P*<5.0×10^−8^ in our analysis.

### Re-analysis of published meta-analyses and selection of genetic variants

In the re-analysis of all the retrieved meta-analyses, the per-allele ORs for individual studies were combined using both fixed effects model and Dersimonian-Laird random effects model meta-analyses. We examined the test for association at the significance level of *P*<2.5×10^−3^ ( = 0.05/20) to correct the multiple testing. Homogeneity across studies was examined by Cochran's *Q* test at the significance level of 0.1. The extent of between-study heterogeneity was quantified by *I* ^2^. *I* ^2^ values over 50% indicate large heterogeneity. All the meta-analyses were performed by using STATA version 11.0.

We selected genetic variants according to the following criteria: First, genetic variants showed evidence of association in the re-analysis of meta-analysis (*P*<2.5×10^−3^) or in the NHGRI GWAS catalog (*P*<0.5×10^−8^). Second, minor allele frequency was larger than 5% in Japanese as Janssens et al. shows low-frequency genetic variants with small effects does not largely affect the predictive ability [105]. The allele frequency in Japanese population was sought in SNP Control Database [106].

In order to introduce possible genetic heterogeneity among ethnic groups into the risk prediction model, we performed subgroup analyses in which ORs per ethnic group were estimated. We used the ethnic group-specific effect into risk prediction model if meta-analysis fulfilled the following criteria: greater than or equal to three independent studies were included and the total number of cases and controls was larger than 2,000 in both target (East Asian) and major (European descent) ethnic groups; the ethnic group-specific OR was statistically significant (*P*<2.5×10^−3^); and heterogeneity in the ethnic group-specific OR between the target and major ethnic groups was statistically significant (*P*<0.05).

### Genetic risk models

We considered three logistic regression models: the HLA model included the *HLA-DRB1* alleles only; the non-HLA model included the selected genetic variants at the non-HLA loci; and the integrative model incorporated both of the *HLA-DRB1* alleles and the genetic variants at the non-HLA loci. In the logistic regression analyses, the genetic risk score is as follows:
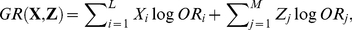
(1)where 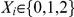 is the number of risk alleles of SNP locus *i*, 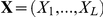 is the genetic profiles of *L* loci genotypes, 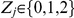 is the indicator variable, indexing the number of each of selected *HLA-DRB1* alleles, 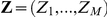 is the profiles showing the subject's *HLA-DRB1* genotype, and the OR for each variant is derived from the re-analysis of meta-analyses. The integrative model was the full model that was expressed as the equation [1], whereas the HLA and the non-HLA models were the reduced models where the first and second terms on the right-hand side of the equation [1] were excluded, respectively.

In order to assess the predictive ability of the models, we used the ROC curve and calculated the AUC [16]. By definition, the AUC is the probability that a randomly selected subject with the disease of interest has a higher score than a randomly selected subject without the disease. When the ROC analyses were implemented, we restricted analyses to subjects with complete genotype data. Thus, 1,231 cases and 1,445 controls were available. We compared the fits of the three models with Akaike's information criterion.

### Simulation study

In most common diseases, the predictive ability of common genetic variants may be suboptimal at the current moment. We therefore performed a simulation study to address how many additional loci should be mapped to establish an acceptable level of genetic risk prediction (AUC = 80.0%).

We assumed two scenarios of allelic architecture of as-yet-discovered genetic variants. First, we assumed the common disease-common variant hypothesis, in which a large proportion of the missing heritability can be explained by common variants [107]. In this model, the per-allele OR was set to be 1.1 or 1.2 and the RAF was set to be 0.1 or 0.3. Second, we assumed that the multiple rare variants with intermediate effects remain undiscovered [14]. In this model, we assumed that the per-allele OR was 2.0 or 3.0 with RAF of 0.01.

To simulate the distribution of RA risks in the general population, we considered the constrained multiplicative model [108]. First, we set ‘base model’, where all the possible combinations of genotypes of *HLA-DRB1* alleles and selected SNPs were included. For the base model, the ORs derived from our case-control association study were used and the allele frequencies in Japanese were obtained from SNP Control Database [106] (shown in Text S1B). Next, we added *N* diallelic loci to the base model. For simplicity, we assumed that the frequency and the effect size of the risk allele at each additional locus are the same as *p* and *OR*, respectively. We assumed that each locus is both in HWE and in linkage equilibrium. We denote *K* as the prevalence of RA and set it to 0.01. Under the rare disease assumption, the relative risk can be approximated by the odds ratio. The risk and the joint probability of multi-locus genotype can be written as the product across loci:
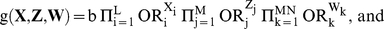
(2)

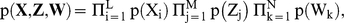
respectively, where *b* is the background risk so that 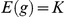, 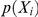 is the probabilities of *X_i_*, and 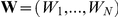 is the genetic profiles of *N*-locus genotypes with 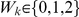 representing the number of risk alleles of additional locus *k*. By the assumption that each additional locus has the same p and OR, the equation [2] can be written down as:
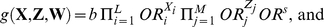
(3)

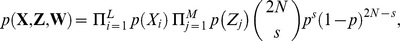
where 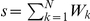. For some genetic profiles with many risk alleles, the risk expressed as the equation [3] can exceed 1. In the constrained multiplicative model, if the risk exceeds 1, the risk is set to 1 [108].

The probability of multi-locus genotype given disease status is:
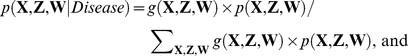


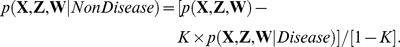
(4)


For an arbitrary cut-off value of *t*, the true and false positive rates are:
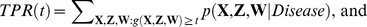


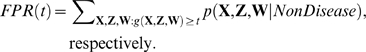
(5)


Given the TPR and FPR at each cut-off value *t*, the ROC curve can be drawn and then the AUC can be calculated by the trapezoid rule [109].

### Network analysis

We assigned selected genetic variants to a single protein-coding gene according to the following hierarchy: coding>intronic>5′UTR>3′UTR>near gene (within 2 kb to 5′ or 0.5 kb to 3′ of a gene)>intergenic. If a selected variant mapped an intergenic region, we sought literature of fine-mapping studies or GWAS of RA and other autoimmune diseases showing evidence of association of variants in higher levels of the hierarchy.

The physical PPI network was constructed using the HPRD database [75], [76]. In PPI networks, vertices are proteins and edges represent a physical interaction between two proteins. We projected the RA-associated genes onto the constructed PPI network and candidate genes were then ranked based on the global distance to the RA-associated genes within the PPI network by using random walk with restart (RWR) algorithm [77]. The RWR algorithm is a powerful tool to measure proximity between vertices on complex network.

In a random walk, starting at some initial ‘seed’ vertices (i.e., proteins encoded by the RA-associated genes), we chose at random an edge that is attached to the current vertex and move along the chosen edge to the linked vertex, and iterate many steps. In the RWR, at each step of the walk we return to the initial seed vertices with the restart probability, *r*. All vertices are ranked by the number of times that the walker visits to corresponding vertices in the process. The outline is described below.

The adjacency matrix **A** of the PPI network is the matrix with elements *A_ij_* as follows: 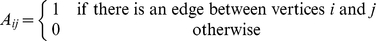. We define the transition probability matrix **M** so that the transition probability *M_ij_* from protein *i* to protein *j* is: 
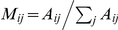
. Let **p** ^(t)^ be a vector whose *i*-th element holds the probability of a random walker being at vertex *i* at step *t* and **p** ^(0)^ be the initial-state probability vector, the probability vector at the step *t*+1 is as follows:
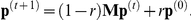



In this study, **p** ^(0)^ was defined as the vector with elements: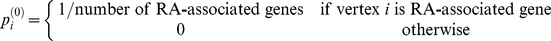. The restart probability *r* was set to be 0.5. We considered the random walker reached a steady-state when the difference between **p** ^(t+1)^ and **p** ^(t)^ (measured by the L1 norm) reached 10^−10^. All the genes in the PPI network were ranked according to the corresponding values in the steady-state probability vector **p** ^(∞)^.

The predictive ability of the network-guided prioritization of genes was tested using leave-one-out cross-validation by omitting each RA-associated gene in turn from initial ‘seed’ vertices and performing the RWR algorithm for the purpose of its own evaluation. The ROC curve was drawn by plotting the TPR versus the FPR for all genes ranked above a sliding ranking threshold.

We define RA-associated network as a subnetwork in which vertices are the RA-associated genes and genes ranked in the top 100 by the RWR algorithm and edges are physical interactions between their products. Functional modules are then explored in the RA-associated network. The overlapping and hierarchical clusters were detected by using the EAGLE algorithm [84]. The functional annotation for the retrieved clusters was performed by using DAVID [85], [86]. We set 9,521 genes on the PPI network from HPRD as the background in enrichment analysis.

## Supporting Information

Figure S1 **Flowchart detailing the exclusion and inclusion criteria and the number of studies excluded and included at each step of the electronic database searches.** A) PubMed, and B) NHGRI GWAS catalog.
(TIF)

Figure S2 **RA-associated network comprising known RA-associated genes and genes ranked in the top 50 by the RWR algorithm and edges are physical interactions between their products.** Nodes are color coded by hierarchical clusters detected by the EAGLE algorithm: CL1, red; CL2; cyan, and CL3, yellow. Overlapped regions between CL1 and CL2 are rendered in green. Node size is based on the ranking in the RWR algorithm.
(TIF)

Figure S3 **RA-associated network comprising known RA-associated genes and genes ranked in the top 150 by the RWR algorithm and edges are physical interactions between their products.** Nodes are color coded by hierarchical clusters detected by the EAGLE algorithm: CL1, red; CL2; cyan, CL3, yellow; and CL4, orange. Overlapped regions between CL1 and CL2, CL1 and CL4, and CL2 and CL4 are rendered in green, pink, and purple, respectively. Node size is based on the ranking in the RWR algorithm.
(TIF)

Figure S4 **Re-consideration on RA-associated network.** The RWR algorithm was re-examined by adding recently discovered 4 genes (*AIRE*, *CD247*, *UBASH3A*, and *ATXN2*). Nodes are color coded by hierarchical clusters detected by the EAGLE algorithm: CL1, red; CL2; cyan, and CL3, yellow. Overlapped regions between CL1 and CL2 are rendered in green. Node size is based on the ranking in the RWR algorithm.
(TIFF)

Table S1 **Result of ratings for 87 abstracts retrieved from PubMed.** The scoring was conducted by independent two authors (Hirofumi Nakaoka and Tailin Cui), which is color-coded in green and blue, respectively. Any disagreement between the two researchers was accommodated by Atsushi Tajima. The final decision is rendered in red.
(DOC)

Table S2 **Result of rating for 54 full-text articles.**
(DOC)

Table S3 **Result of screening of extracted data from 51 full-text articles.**
(DOC)

Table S4 **Re-analysis of meta-analyses addressing genetic associations with RA risk.**
(DOC)

Table S5 **Assignment of a single gene to genetic variants associated with RA and the allele frequencies in European and Japanese.**
(DOC)

Table S6 **Ethnic group-specific analysis of published meta-analyses of genetic associations with RA risk.** The SNPs in which the heterogeneity in the ORs between European and East Asian populations are significant are highlighted in yellow.
(DOC)

Table S7 **Genotype counts for six** ***HLA-DRB1*** **alleles and 15 SNPs.**
(DOC)

Table S8 **Association analysis of RF and anti-CCP positive RA patients versus control subjects with selected genetic variants.**
(DOC)

Table S9 **GO and KEGG annotations for three clusters in RA-associated network comprising RA-associated genes and genes ranked in the top 50 by the RWR algorithm.**
(DOC)

Table S10 **GO and KEGG annotations for three clusters in RA-associated network comprising RA-associated genes and genes ranked in the top 150 by the RWR algorithm.**
(DOC)

Table S11 **Re-consideration on RA-associated network.** The RWR algorithm was re-examined by adding recently discovered 4 genes (*AIRE*, *CD247*, *UBASH3A*, and *ATXN2*). GO and KEGG annotations for three clusters in RA-associated network comprising RA-associated genes and genes ranked in the top 100 by the RWR algorithm.
(DOC)

Text S1 **Supplementary methods.**
(DOC)
