# Supplementary figures and images for: A Systems Genetics Approach Provides a Bridge from Discovered Genetic Variants to Biological Pathways in Rheumatoid Arthritis
Source: PLoS One. 2011 Sep 28;6(9):e25389. doi: 10.1371/journal.pone.0025389 (PMC3182219; doi:10.1371/journal.pone.0025389)

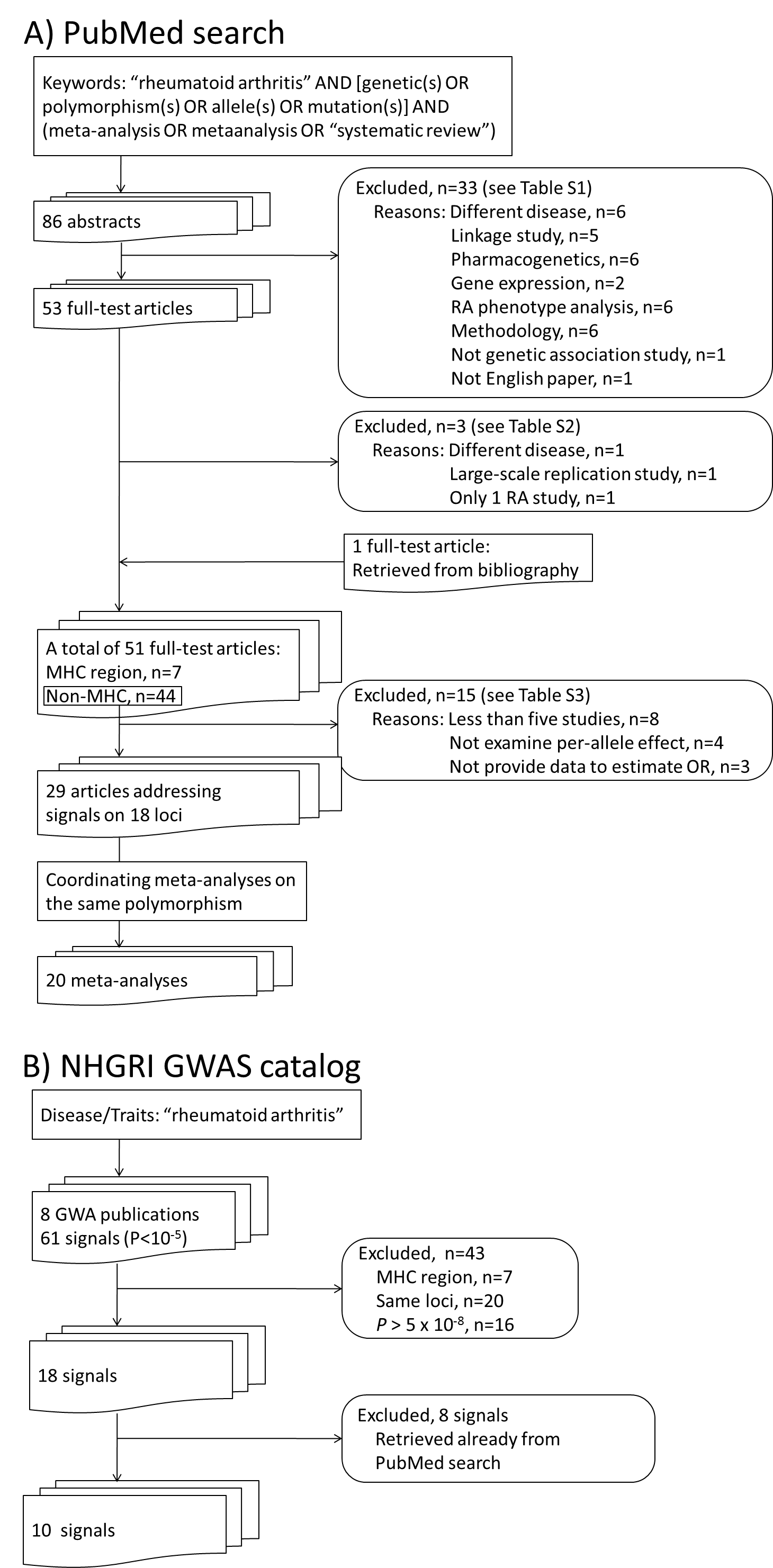

Supplement: Figure S1 — Flowchart detailing the exclusion and inclusion criteria and the number of studies excluded and included at each step of the electronic database searches. A) PubMed, and B) NHGRI GWAS catalog. (TIF) [file pone.0025389.s001.tif]

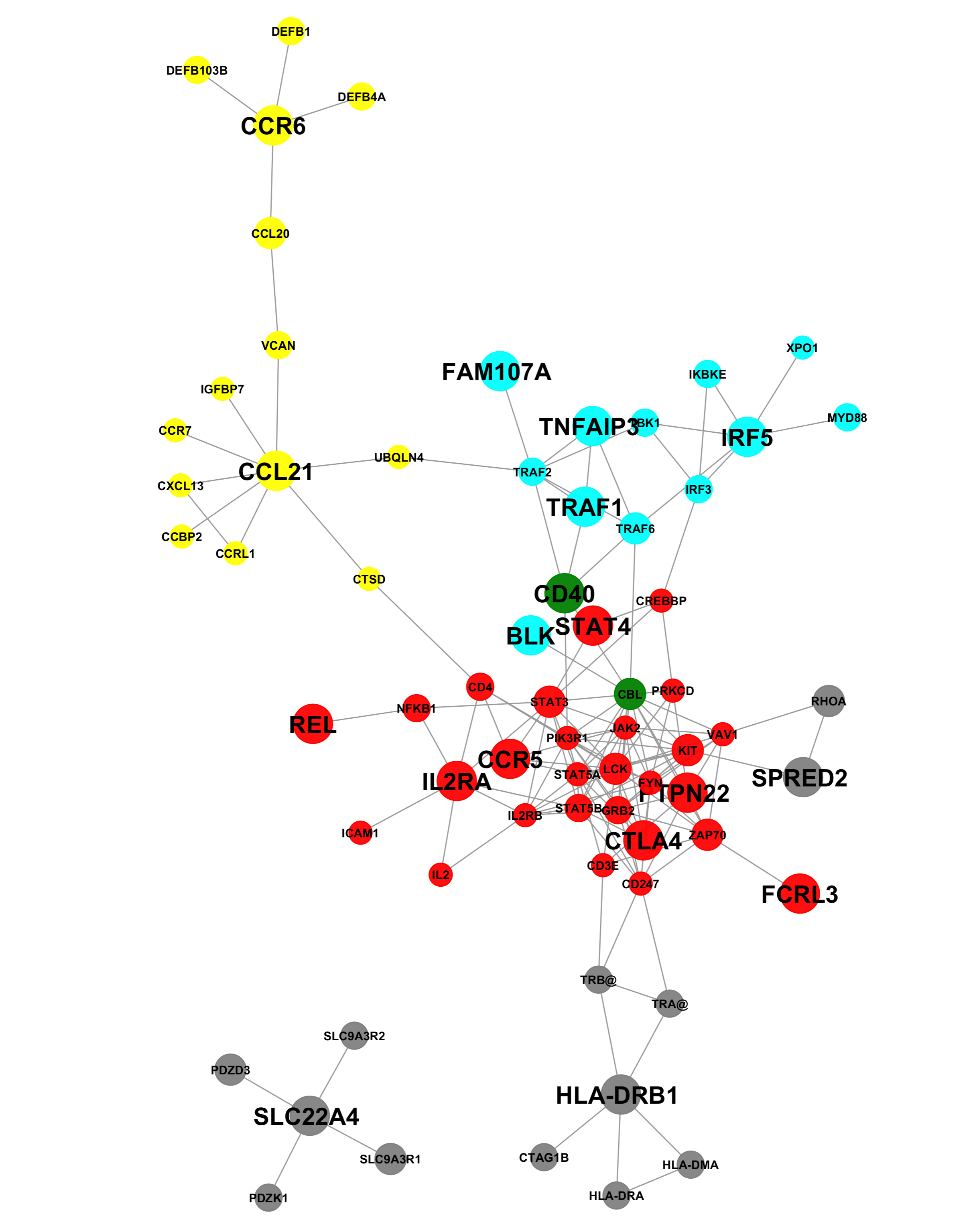

Supplement: Figure S2 — RA-associated network comprising known RA-associated genes and genes ranked in the top 50 by the RWR algorithm and edges are physical interactions between their products. Nodes are color coded by hierarchical clusters detected by the EAGLE algorithm: CL1, red; CL2; cyan, and CL3, yellow. Overlapped regions between CL1 and CL2 are rendered in green. Node size is based on the ranking in the RWR algorithm. (TIF) [file pone.0025389.s002.tif]

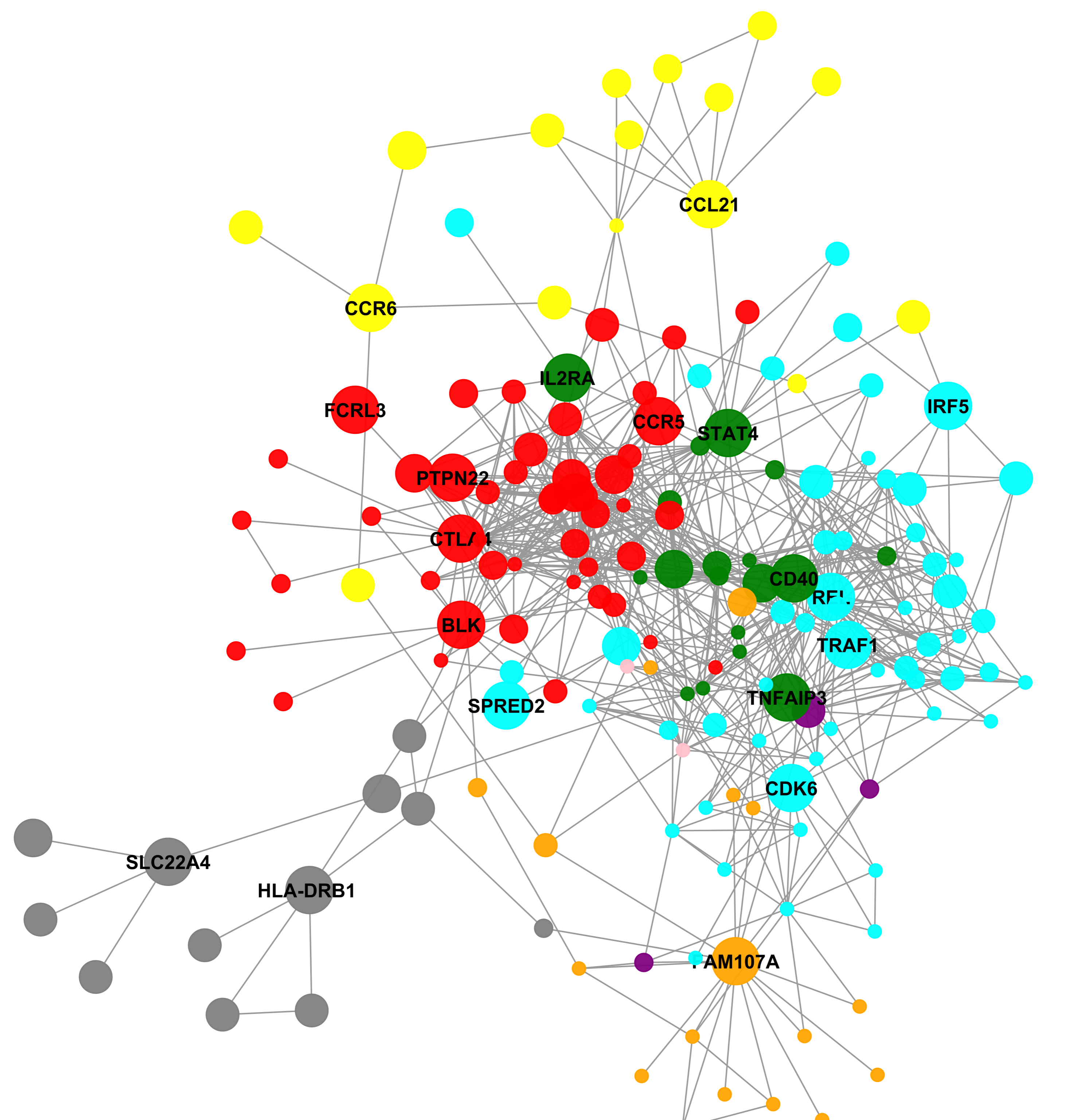

Supplement: Figure S3 — RA-associated network comprising known RA-associated genes and genes ranked in the top 150 by the RWR algorithm and edges are physical interactions between their products. Nodes are color coded by hierarchical clusters detected by the EAGLE algorithm: CL1, red; CL2; cyan, CL3, yellow; and CL4, orange. Overlapped regions between CL1 and CL2, CL1 and CL4, and CL2 and CL4 are rendered in green, pink, and purple, respectively. Node size is based on the ranking in the RWR algorithm. (TIF) [file pone.0025389.s003.tif]

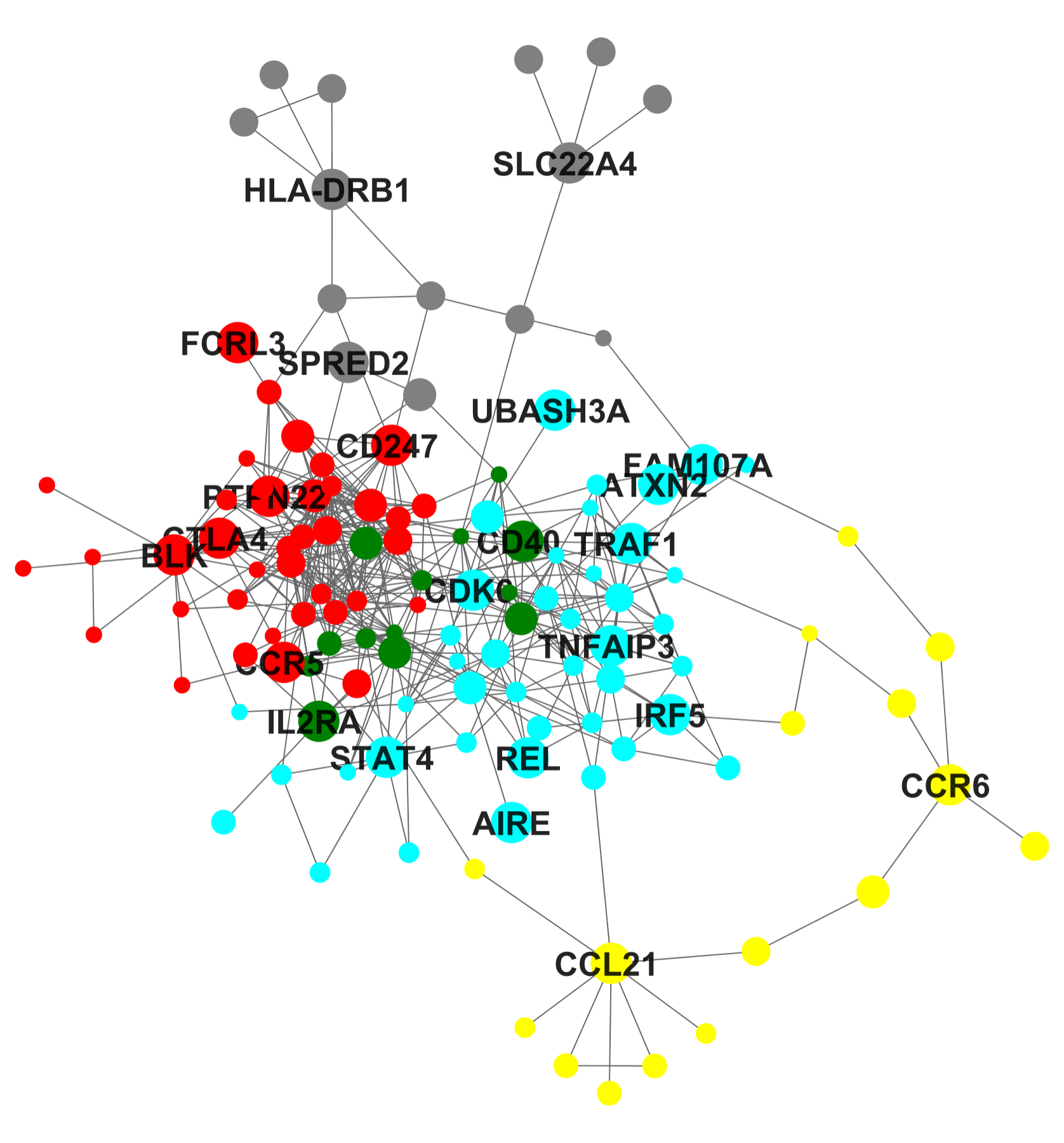

Supplement: Figure S4 — Re-consideration on RA-associated network. The RWR algorithm was re-examined by adding recently discovered 4 genes (AIRE, CD247, UBASH3A, and ATXN2). Nodes are color coded by hierarchical clusters detected by the EAGLE algorithm: CL1, red; CL2; cyan, and CL3, yellow. Overlapped regions between CL1 and CL2 are rendered in green. Node size is based on the ranking in the RWR algorithm. (TIFF) [file pone.0025389.s004.tif]
