# Supplementary material for: A Systems Genetics Approach Provides a Bridge from Discovered Genetic Variants to Biological Pathways in Rheumatoid Arthritis
Source: PLoS One. 2011 Sep 28;6(9):e25389. doi: 10.1371/journal.pone.0025389 (PMC3182219; doi:10.1371/journal.pone.0025389)
Supplement: Table S1 — Result of ratings for 87 abstracts retrieved from PubMed. The scoring was conducted by independent two authors (Hirofumi Nakaoka and Tailin Cui), which is color-coded in green and blue, respectively. Any disagreement between the two researchers was accommodated by Atsushi Tajima. The final decision is rendered in red. (DOC) [file pone.0025389.s005.doc]

**Table S1.** Result of ratings for 87 abstracts retrieved from PubMed. The scoring was conducted by independent two authors (Hirofumi Nakaoka and Tailin Cui), which is color-coded in green and blue, respectively. Any disagreement between the two researchers was accommodated by Atsushi Tajima. The final decision is rendered in red.

| Ref  ID^A^ | First Author | Data  extraction | | | Different  disease | | | Linkage  study | | | Pharmaco  genomics | | | Gene  expression | | | RA  phenotype  (case only) | | | Methodology | | | Not genetic  association  study | | | Not English  language paper | | |
| --- | --- | --- | --- | --- | --- | --- | --- | --- | --- | --- | --- | --- | --- | --- | --- | --- | --- | --- | --- | --- | --- | --- | --- | --- | --- | --- | --- | --- |
| 1 | Alizadeh | ✓ | ✓ | ✓ |  |  |  |  |  |  |  |  |  |  |  |  |  |  |  |  |  |  |  |  |  |  |  |  |
| 2 | Barnetche | ✓ | ✓ | ✓ |  |  |  |  |  |  |  |  |  |  |  |  |  |  |  |  |  |  |  |  |  |  |  |  |
| 3 | Begovich | ✓ | ✓ | ✓ |  |  |  |  |  |  |  |  |  |  |  |  |  |  |  |  |  |  |  |  |  |  |  |  |
| 4 | Briollais |  |  |  |  |  |  |  |  |  |  |  |  |  |  |  |  |  |  | ✓ | ✓ | ✓ |  |  |  |  |  |  |
| 5 | Bronson | ✓ | ✓ | ✓ |  |  |  |  |  |  |  |  |  |  |  |  |  |  |  |  |  |  |  |  |  |  |  |  |
| 6 | Burr | ✓ | ✓ | ✓ |  |  |  |  |  |  |  |  |  |  |  |  |  |  |  |  |  |  |  |  |  |  |  |  |
| 7 | Carr |  |  |  | ✓ | ✓ | ✓ |  |  |  |  |  |  |  |  |  |  |  |  |  |  |  |  |  |  |  |  |  |
| 8 | Chang | ✓ | ✓ | ✓ |  |  |  |  |  |  |  |  |  |  |  |  |  |  |  |  |  |  |  |  |  |  |  |  |
| 9 | Chelala | ✓ | ✓ | ✓ |  |  |  |  |  |  |  |  |  |  |  |  |  |  |  |  |  |  |  |  |  |  |  |  |
| 10 | Choi |  |  |  |  |  |  | ✓ | ✓ | ✓ |  |  |  |  |  |  |  |  |  |  |  |  |  |  |  |  |  |  |
| 11 | Coenen | ✓ | ✓ | ✓ |  |  |  |  |  |  |  |  |  |  |  |  |  |  |  |  |  |  |  |  |  |  |  |  |
| 12 | Daha | ✓ | ✓ | ✓ |  |  |  |  |  |  |  |  |  |  |  |  |  |  |  |  |  |  |  |  |  |  |  |  |
| 13 | Delgado-Vega | ✓ | ✓ | ✓ |  |  |  |  |  |  |  |  |  |  |  |  |  |  |  |  |  |  |  |  |  |  |  |  |
| 14 | Dieguez-Gonzalez | ✓ | ✓ | ✓ |  |  |  |  |  |  |  |  |  |  |  |  |  |  |  |  |  |  |  |  |  |  |  |  |
| 15 | Etzel |  |  |  |  |  |  | ✓ |  | ✓ |  |  |  |  |  |  |  |  |  |  | ✓ |  |  |  |  |  |  |  |
| 16 | Eyre | ✓ | ✓ | ✓ |  |  |  |  |  |  |  |  |  |  |  |  |  |  |  |  |  |  |  |  |  |  |  |  |
| 17 | Eyre | ✓ | ✓ | ✓ |  |  |  |  |  |  |  |  |  |  |  |  |  |  |  |  |  |  |  |  |  |  |  |  |
| 18 | Festen |  |  |  | ✓ | ✓ | ✓ |  |  |  |  |  |  |  |  |  |  |  |  |  |  |  |  |  |  |  |  |  |
| 19 | Fisher |  |  |  |  |  |  |  |  |  | ✓ | ✓ | ✓ |  |  |  |  |  |  |  |  |  |  |  |  |  |  |  |
| 20 | Fisher |  |  |  |  |  |  | ✓ | ✓ | ✓ |  |  |  |  |  |  |  |  |  |  |  |  |  |  |  |  |  |  |
| 21 | Glinskii |  |  |  |  |  |  |  |  |  |  |  |  | ✓ | ✓ | ✓ |  |  |  |  |  |  |  |  |  |  |  |  |
| 22 | Gorman |  |  |  |  |  |  |  |  |  |  |  |  |  |  |  | ✓ | ✓ | ✓ |  |  |  |  |  |  |  |  |  |
| 23 | Gorman |  |  |  |  |  |  |  |  |  |  |  |  |  |  |  | ✓ | ✓ | ✓ |  |  |  |  |  |  |  |  |  |
| 24 | Gorman |  |  |  |  |  |  |  |  |  |  |  |  |  |  |  | ✓ | ✓ | ✓ |  |  |  |  |  |  |  |  |  |
| 25 | Gorman |  |  |  |  |  |  |  |  |  |  |  |  |  |  |  | ✓ | ✓ | ✓ |  |  |  |  |  |  |  |  |  |
| 26 | Graham |  |  |  | ✓ | ✓ | ✓ |  |  |  |  |  |  |  |  |  |  |  |  |  |  |  |  |  |  |  |  |  |
| 27 | Han | ✓ | ✓ | ✓ |  |  |  |  |  |  |  |  |  |  |  |  |  |  |  |  |  |  |  |  |  |  |  |  |
| 28 | Han | ✓ | ✓ | ✓ |  |  |  |  |  |  |  |  |  |  |  |  |  |  |  |  |  |  |  |  |  |  |  |  |
| 29 | Harrison | ✓ | ✓ | ✓ |  |  |  |  |  |  |  |  |  |  |  |  |  |  |  |  |  |  |  |  |  |  |  |  |
| 30 | Hinks |  |  |  | ✓ | ✓ | ✓ |  |  |  |  |  |  |  |  |  |  |  |  |  |  |  |  |  |  |  |  |  |
| 31 | Hollis-Moffatt | ✓ | ✓ | ✓ |  |  |  |  |  |  |  |  |  |  |  |  |  |  |  |  |  |  |  |  |  |  |  |  |
| 32 | Hollis-Moffatt | ✓ | ✓ | ✓ |  |  |  |  |  |  |  |  |  |  |  |  |  |  |  |  |  |  |  |  |  |  |  |  |
| 33 | Ioannidis | ✓ | ✓ | ✓ |  |  |  |  |  |  |  |  |  |  |  |  |  |  |  |  |  |  |  |  |  |  |  |  |
| 34 | Issekutz |  | ✓ |  |  |  |  |  |  |  |  |  |  |  |  |  |  |  |  |  |  |  |  |  |  | ✓ |  | ✓ |
| 35 | Iwamoto | ✓ | ✓ | ✓ |  |  |  |  |  |  |  |  |  |  |  |  |  |  |  |  |  |  |  |  |  |  |  |  |
| 36 | Ji | ✓ | ✓ | ✓ |  |  |  |  |  |  |  |  |  |  |  |  |  |  |  |  |  |  |  |  |  |  |  |  |
| 37 | Jun | ✓ | ✓ | ✓ |  |  |  |  |  |  |  |  |  |  |  |  |  |  |  |  |  |  |  |  |  |  |  |  |
| 38 | Katchamart |  |  |  |  |  |  |  |  |  |  |  |  |  |  |  | ✓ | ✓ | ✓ |  |  |  |  |  |  |  |  |  |
| 39 | Kim | ✓ | ✓ | ✓ |  |  |  |  |  |  |  |  |  |  |  |  |  |  |  |  |  |  |  |  |  |  |  |  |
| 40 | Kurreeman |  |  |  | ✓ | ✓ | ✓ |  |  |  |  |  |  |  |  |  |  |  |  |  |  |  |  |  |  |  |  |  |
| 41 | Lee | ✓ | ✓ | ✓ |  |  |  |  |  |  |  |  |  |  |  |  |  |  |  |  |  |  |  |  |  |  |  |  |
| 42 | Lee |  |  |  |  |  |  |  |  |  | ✓ | ✓ | ✓ |  |  |  |  |  |  |  |  |  |  |  |  |  |  |  |
| 43 | Lee | ✓ | ✓ | ✓ |  |  |  |  |  |  |  |  |  |  |  |  |  |  |  |  |  |  |  |  |  |  |  |  |
| 44 | Lee | ✓ | ✓ | ✓ |  |  |  |  |  |  |  |  |  |  |  |  |  |  |  |  |  |  |  |  |  |  |  |  |
| 45 | Lee | ✓ | ✓ | ✓ |  |  |  |  |  |  |  |  |  |  |  |  |  |  |  |  |  |  |  |  |  |  |  |  |
| 46 | Lee | ✓ | ✓ | ✓ |  |  |  |  |  |  |  |  |  |  |  |  |  |  |  |  |  |  |  |  |  |  |  |  |
| 47 | Lee |  |  |  |  |  |  |  |  |  | ✓ | ✓ | ✓ |  |  |  |  |  |  |  |  |  |  |  |  |  |  |  |
| 48 | Lee | ✓ | ✓ | ✓ |  |  |  |  |  |  |  |  |  |  |  |  |  |  |  |  |  |  |  |  |  |  |  |  |
| 49 | Lee |  |  |  |  |  |  |  |  |  | ✓ | ✓ | ✓ |  |  |  |  |  |  |  |  |  |  |  |  |  |  |  |
| 50 | Lee | ✓ | ✓ | ✓ |  |  |  |  |  |  |  |  |  |  |  |  |  |  |  |  |  |  |  |  |  |  |  |  |
| 51 | Lee | ✓ | ✓ | ✓ |  |  |  |  |  |  |  |  |  |  |  |  |  |  |  |  |  |  |  |  |  |  |  |  |
| 52 | Lei | ✓ | ✓ | ✓ |  |  |  |  |  |  |  |  |  |  |  |  |  |  |  |  |  |  |  |  |  |  |  |  |
| 53 | Lin |  |  |  |  |  |  |  |  |  |  |  |  |  |  |  |  |  |  | ✓ | ✓ | ✓ |  |  |  |  |  |  |
| 54 | Linden |  |  |  |  |  |  |  |  |  |  |  |  |  |  |  | ✓ | ✓ | ✓ |  |  |  |  |  |  |  |  |  |
| 55 | Lindner | ✓ | ✓ | ✓ |  |  |  |  |  |  |  |  |  |  |  |  |  |  |  |  |  |  |  |  |  |  |  |  |
| 56 | Maiti | ✓ | ✓ | ✓ |  |  |  |  |  |  |  |  |  |  |  |  |  |  |  |  |  |  |  |  |  |  |  |  |
| 57 | McKinney | ✓ | ✓ | ✓ |  |  |  |  |  |  |  |  |  |  |  |  |  |  |  |  |  |  |  |  |  |  |  |  |
| 58 | Merriman |  |  |  |  |  |  | ✓ | ✓ | ✓ |  |  |  |  |  |  |  |  |  |  |  |  |  |  |  |  |  |  |
| 59 | Nishino | ✓ |  |  |  | ✓ | ✓ |  |  |  |  |  |  |  |  |  |  |  |  |  |  |  |  |  |  |  |  |  |
| 60 | O'Rielly |  |  |  |  |  |  |  |  |  | ✓ | ✓ | ✓ |  |  |  |  |  |  |  |  |  |  |  |  |  |  |  |
| 61 | Okada | ✓ | ✓ | ✓ |  |  |  |  |  |  |  |  |  |  |  |  |  |  |  |  |  |  |  |  |  |  |  |  |
| 62 | Orozco | ✓ | ✓ | ✓ |  |  |  |  |  |  |  |  |  |  |  |  |  |  |  |  |  |  |  |  |  |  |  |  |
| 63 | Orozco | ✓ | ✓ | ✓ |  |  |  |  |  |  |  |  |  |  |  |  |  |  |  |  |  |  |  |  |  |  |  |  |
| 64 | Patsopoulos | ✓ | ✓ | ✓ |  |  |  |  |  |  |  |  |  |  |  |  |  |  |  |  |  |  |  |  |  |  |  |  |
| 65 | Pavy |  |  |  |  |  |  |  |  |  | ✓ | ✓ | ✓ |  |  |  |  |  |  |  |  |  |  |  |  |  |  |  |
| 66 | Plant | ✓ | ✓ | ✓ |  |  |  |  |  |  |  |  |  |  |  |  |  |  |  |  |  |  |  |  |  |  |  |  |
| 67 | Plant | ✓ | ✓ | ✓ |  |  |  |  |  |  |  |  |  |  |  |  |  |  |  |  |  |  |  |  |  |  |  |  |
| 68 | Plenge | ✓ | ✓ | ✓ |  |  |  |  |  |  |  |  |  |  |  |  |  |  |  |  |  |  |  |  |  |  |  |  |
| 69 | Prahalad | ✓ | ✓ | ✓ |  |  |  |  |  |  |  |  |  |  |  |  |  |  |  |  |  |  |  |  |  |  |  |  |
| 70 | Raychaudhuri | ✓ | ✓ | ✓ |  |  |  |  |  |  |  |  |  |  |  |  |  |  |  |  |  |  |  |  |  |  |  |  |
| 71 | Raychaudhuri | ✓ | ✓ | ✓ |  |  |  |  |  |  |  |  |  |  |  |  |  |  |  |  |  |  |  |  |  |  |  |  |
| 72 | Segurado |  |  |  |  |  |  | ✓ | ✓ | ✓ |  |  |  |  |  |  |  |  |  |  |  |  |  |  |  |  |  |  |
| 73 | Silva |  |  |  |  |  |  |  |  |  |  |  |  | ✓ | ✓ | ✓ |  |  |  |  |  |  |  |  |  |  |  |  |
| 74 | Somers |  |  |  |  |  |  |  |  |  |  |  |  |  |  |  |  | ✓ |  |  |  |  | ✓ |  | ✓ |  |  |  |
| 75 | Stahl | ✓ | ✓ | ✓ |  |  |  |  |  |  |  |  |  |  |  |  |  |  |  |  |  |  |  |  |  |  |  |  |
| 76 | Suarez-Gestal | ✓ | ✓ | ✓ |  |  |  |  |  |  |  |  |  |  |  |  |  |  |  |  |  |  |  |  |  |  |  |  |
| 77 | Takata | ✓ | ✓ | ✓ |  |  |  |  |  |  |  |  |  |  |  |  |  |  |  |  |  |  |  |  |  |  |  |  |
| 78 | Tapper |  |  |  |  | ✓ |  |  |  |  |  | ✓ |  |  |  |  |  |  |  | ✓ |  | ✓ |  |  |  |  |  |  |
| 79 | Telenti |  | ✓ |  |  |  |  |  |  |  | ✓ |  |  |  |  |  |  |  |  | ✓ |  | ✓ | ✓ |  |  |  |  |  |
| 80 | Thabet | ✓ | ✓ | ✓ |  |  |  |  |  |  |  |  |  |  |  |  |  |  |  |  |  |  |  |  |  |  |  |  |
| 81 | Thomas |  |  |  |  |  |  |  |  |  |  |  |  |  |  |  |  |  |  | ✓ | ✓ | ✓ |  |  |  |  |  |  |
| 82 | Wheeler | ✓ |  | ✓ |  | ✓ |  |  |  |  |  | ✓ |  |  |  |  |  |  |  |  |  |  |  |  |  |  |  |  |
| 83 | Williams | ✓ | ✓ | ✓ |  |  |  |  |  |  |  |  |  |  |  |  |  |  |  |  |  |  |  |  |  |  |  |  |
| 84 | Woude | ✓ | ✓ | ✓ |  |  |  |  |  |  |  |  |  |  |  |  |  |  |  |  |  |  |  |  |  |  |  |  |
| 85 | Yu | ✓ | ✓ | ✓ |  |  |  |  |  |  |  |  |  |  |  |  |  |  |  |  |  |  |  |  |  |  |  |  |
| 86 | Zintzaras |  |  |  |  |  |  |  |  |  |  |  |  |  |  |  |  |  |  | ✓ | ✓ | ✓ |  |  |  |  |  |  |
| 87 | Fernando | ✓ | ✓ | ✓ |  |  |  |  |  |  |  |  |  |  |  |  |  |  |  |  |  |  |  |  |  |  |  |  |

^A^ Ref ID corresponds to the reference number of Text S1.
