# Supplementary material for: A Systems Genetics Approach Provides a Bridge from Discovered Genetic Variants to Biological Pathways in Rheumatoid Arthritis
Source: PLoS One. 2011 Sep 28;6(9):e25389. doi: 10.1371/journal.pone.0025389 (PMC3182219; doi:10.1371/journal.pone.0025389)
Supplement: Table S3 — Result of screening of extracted data from 51 full-text articles. (DOC) [file pone.0025389.s007.doc]

**Table S3.** Result of screening of extracted data from 51 full-text articles.

| Ref  ID^A^ | First author | Year | Suffice  inclusion criteria | Reason for exclusion |
| --- | --- | --- | --- | --- |
| 1 | Alizadeh | 2007 |  | Not examine per-allele effect |
| 2 | Barnetche | 2008 |  | HLA-DRB1 |
| 3 | Begovich | 2007 | ✓ |  |
| 5 | Bronson | 2008 |  | Not examine per-allele effect |
| 6 | Burr | 2010 | ✓ |  |
| 8 | Chang | 2010 |  | Not examine per-allele effect |
| 11 | Coenen | 2009 |  | Less than five studies |
| 12 | Daha | 2009 | ✓ |  |
| 13 | Delgado-Vega | 2007 |  | HLA-DRB1 |
| 14 | Dieguez-Gonzalez | 2008 | ✓ |  |
| 17 | Eyre | 2010 |  | Less than five studies |
| 27 | Han | 2005 | ✓ |  |
| 28 | Han | 2009 | ✓ |  |
| 29 | Harrison | 2008 | ✓ |  |
| 31 | Hollis-Moffatt | 2009 |  | Less than five studies |
| 32 | Hollis-Moffatt | 2009 |  | Less than five studies |
| 33 | Ioannidis | 2002 |  | HLA-DRB1 |
| 35 | Iwamoto | 2006 | ✓ |  |
| 36 | Ji | 2010 | ✓ |  |
| 37 | Jun | 2007 |  | HLA-DRB1 |
| 39 | Kim | 2008 |  | Less than five studies |
| 41 | Lee | 2009 |  | Less than five studies |
| 43 | Lee | 2009 | ✓ |  |
| 44 | Lee | 2008 | ✓ |  |
| 45 | Lee | 2007 | ✓ |  |
| 46 | Lee | 2007 | ✓ |  |
| 48 | Lee | 2007 | ✓ |  |
| 50 | Lee | 2010 | ✓ |  |
| 51 | Lee | 2010 | ✓ |  |
| 52 | Lei | 2005 | ✓ |  |
| 55 | Lindner | 2007 | ✓ |  |
| 56 | Maiti | 2010 |  | Less than five studies |
| 57 | McKinney | 2010 |  | Less than five studies |
| 61 | Okada | 2008 | ✓ |  |
| 62 | Orozco | 2008 | ✓ |  |
| 63 | Orozco | 2010 | ✓ |  |
| 64 | Patsopoulos | 2010 | ✓ |  |
| 66 | Plant | 2009 | ✓ |  |
| 67 | Plant | 2010 |  | Not provide adequate data to calculate odds ratio for each of the included studies |
| 68 | Plenge | 2005 | ✓ |  |
| 69 | Prahalad | 2006 | ✓ |  |
| 70 | Raychaudhuri | 2008 | ✓ |  |
| 71 | Raychaudhuri | 2009 |  | Not provide adequate data to calculate odds ratio for each of the included studies |
| 75 | Stahl | 2010 |  | Not provide adequate data to calculate odds ratio for each of the included studies |
| 76 | Suarez-Gestal | 2009 | ✓ |  |
| 77 | Takata | 2008 | ✓ |  |
| 80 | Thabet | 2007 |  | Not examine per-allele effect |
| 82 | Wheeler | 2007 | ✓ |  |
| 83 | Williams | 1995 |  | HLA-DRB1 |
| 84 | Woude | 2010 |  | HLA-DRB1 |
| 87 | Fernando | 2008 |  | MHC region including HLA-DRB1 |

^A^ Ref ID corresponds to the reference number of Text S1.
