# Supplementary material for: A Systems Genetics Approach Provides a Bridge from Discovered Genetic Variants to Biological Pathways in Rheumatoid Arthritis
Source: PLoS One. 2011 Sep 28;6(9):e25389. doi: 10.1371/journal.pone.0025389 (PMC3182219; doi:10.1371/journal.pone.0025389)
Supplement: Table S4 — Re-analysis of meta-analyses addressing genetic associations with RA risk. (DOC) [file pone.0025389.s008.doc]

**Table S4.** Re-analysis of meta-analyses addressing genetic associations with RA risk.

|  |  |  | Sample size | |  |  | Fixed effects model | | |  | Random effects model | | |
| --- | --- | --- | --- | --- | --- | --- | --- | --- | --- | --- | --- | --- | --- |
| Gene | SNP ID | Studies | Cases | Controls | *I*^2^ (%) | *P_hetero_* | OR | 95% CI | *P* |  | OR | 95% CI | *P* |
| *STAT4* | rs7574865 | 17 | 18,405 | 19,181 | 48.7 | 0.013 | 1.232 | 1.191-1.274 | 4.0×10^-34^ |  | 1.254 | 1.194-1.317 | 1.7×10^-19^ |
| *FCRL3* | rs7528684 | 14 | 9,756 | 9,012 | 50.8 | 0.015 | 1.083 | 1.038-1.129 | 2.2×10^-4^ |  | 1.090 | 1.025-1.160 | 6.1×10^-3^ |
| *TNF-α* | rs1800629 | 13 | 1,462 | 2,306 | 81.6 | 2.6×10^-9^ | 0.952 | 0.824-1.100 | 0.51 |  | 0.999 | 0.697-1.432 | 0.99 |
| *TRAF1-C5* | rs3761847 | 13 | 10,979 | 12,959 | 83.4 | 1.1×10^-10^ | 1.129 | 1.088-1.171 | 9.2×10^-11^ |  | 1.183 | 1.072-1.306 | 8.1×10^-4^ |
| *CCL21* | rs2812378 | 12 | 11,011 | 21,194 | 0.0 | 0.60 | 1.109 | 1.069-1.150 | 2.7×10^-8^ |  | 1.109 | 1.069-1.150 | 2.7×10^-8^ |
| *CD40* | rs4810485 | 12 | 10,988 | 21,292 | 7.5 | 0.37 | 0.868 | 0.833-0.904 | 9.8×10^-12^ |  | 0.868 | 0.831-0.907 | 2.6×10^-10^ |
| *CDK6* | rs42041 | 12 | 11,023 | 21,207 | 34.9 | 0.11 | 1.082 | 1.040-1.125 | 9.6×10^-5^ |  | 1.081 | 1.025-1.141 | 4.2×10^-3^ |
| *PADI4* | rs2240340 | 12 | 12,577 | 18,745 | 77.8 | 7.6×10^-7^ | 1.091 | 1.053-1.131 | 2.0×10^-6^ |  | 1.161 | 1.065-1.266 | 7.0×10^-4^ |
| *PTPN22* | rs2476601 | 12 | 6,950 | 7,887 | 16.7 | 0.28 | 1.638 | 1.523-1.761 | 2.0×10^-40^ |  | 1.650 | 1.520-1.791 | 7.3×10^-33^ |
| *SLC22A4* | rs2073838 | 11 | 12,717 | 10,499 | 14.3 | 0.31 | 1.114 | 1.054-1.178 | 1.3×10^-4^ |  | 1.109 | 1.043-1.180 | 9.7×10^-4^ |
| *IL1B* | rs16944 | 11 | 3,462 | 3,145 | 8.5 | 0.36 | 1.073 | 0.998-1.154 | 0.058 |  | 1.075 | 0.992-1.164 | 0.077 |
| *IL1B* | rs1143634 | 10 | 1,654 | 1,723 | 64.8 | 2.4×10^-3^ | 0.832 | 0.729-0.948 | 6.0×10^-3^ |  | 0.798 | 0.628-1.015 | 0.066 |
| *IRF5* | rs2004640 | 10 | 6,566 | 5,340 | 30.3 | 0.17 | 0.903 | 0.856-0.952 | 1.4×10^-4^ |  | 0.900 | 0.843-0.961 | 1.7×10^-3^ |
| *FCGR3A* | rs396991 | 9 | 2,422 | 2,490 | 63.6 | 5.0×10^-3^ | 1.074 | 0.986-1.170 | 0.10 |  | 1.037 | 0.890-1.208 | 0.65 |
| *CTLA4* | rs3087243 | 8 | 6,329 | 5,330 | 29.0 | 0.20 | 0.894 | 0.845-0.945 | 8.5×10^-5^ |  | 0.891 | 0.832-0.954 | 9.7×10^-4^ |
| *TNFAIP3-OLIG3* | rs6920220 | 7 | 8,637 | 9,888 | 0.0 | 0.81 | 1.262 | 1.201-1.326 | 3.9×10^-20^ |  | 1.262 | 1.201-1.326 | 3.9×10^-20^ |
| *CCR5* | rs333 | 7 | 2,768 | 3,441 | 45.9 | 0.085 | 0.741 | 0.647-0.849 | 1.5×10^-5^ |  | 0.723 | 0.589-0.886 | 1.8×10^-3^ |
| *TNFRSF1B* | rs1061622 | 6 | 3,393 | 2,071 | 51.0 | 0.070 | 0.985 | 0.898-1.081 | 0.75 |  | 1.030 | 0.885-1.200 | 0.70 |
| *BANK1* | rs17266594 | 5 | 3,550 | 3,960 | 21.3 | 0.28 | 0.970 | 0.901-1.044 | 0.42 |  | 0.957 | 0.875-1.047 | 0.34 |
| *TNFAIP3-OLIG3* | rs10499194 | 5 | 4,533 | 7,399 | 67.5 | 0.015 | 0.820 | 0.771-0.871 | 1.8×10^-10^ |  | 0.802 | 0.716-0.898 | 1.3×10^-4^ |
