# Supplementary material for: A Systems Genetics Approach Provides a Bridge from Discovered Genetic Variants to Biological Pathways in Rheumatoid Arthritis
Source: PLoS One. 2011 Sep 28;6(9):e25389. doi: 10.1371/journal.pone.0025389 (PMC3182219; doi:10.1371/journal.pone.0025389)
Supplement: Table S5 — Assignment of a single gene to genetic variants associated with RA and the allele frequencies in European and Japanese. (DOC) [file pone.0025389.s009.doc]

**Table S5.** Assignment of a single gene to genetic variants associated with RA and the allele frequencies in European and Japanese.

| SNP ID | Variant  allele | Effect | Assigned gene | European | Japanese |
| --- | --- | --- | --- | --- | --- |
| rs7574865 | T | Intronic | *STAT4* | 0.230 | 0.355 |
| rs7528684 | G | Upstream | *FCRL3* | 0.487 | 0.427 |
| rs3761847 | G | 5'UTR | *TRAF1* | 0.478 | 0.451 |
| rs2812378 | G | Upstream | *CCL21* | 0.346 | 0.049 |
| rs4810485 | T | Intronic | *CD40* | 0.248 | 0.406 |
| rs42041 | G | Intronic | *CDK6* | 0.314 | 0.019 |
| rs2240340 | T | Intronic | *PADI4* | 0.415 | 0.390 |
| rs2476601 | A | Nonsynonymous | *PTPN22* | 0.117 | 0.032 |
| rs2073838 | A | Intronic | *SLC22A4* | 0.093 | 0.326 |
| rs2004640 | G | Essential splice site, Intronic | *IRF5* | 0.466 | 0.678 |
| rs3087243 | A | Downstream | *CTLA4* | 0.460 | 0.258 |
| rs6920220 | A | Intergenic | *TNFAIP3* | 0.165 | 0.002 |
| rs333 | delta32 | Frameshift | *CCR5* | 0.094 | 0.000 |
| rs10499194 | T | Intergenic | *TNFAIP3* | 0.177 | 0.091 |
| rs3093024 | A | Intronic | *CCR6* | 0.407 | 0.431 |
| rs874040 | C | Intergenic | *SEL1L3-RBPJ* | 0.292 | 0.005 |
| rs11676922 | T | Intergenic | *AFF3-LONRF2* | 0.451 | 0.500 |
| rs13017599 | A | Intronic | *REL* | 0.381 | 0.022 |
| rs6859219 | A | Intronic | *ANKRD55* | 0.170 | 0.023 |
| rs934734 | G | Intronic | *SPRED2* | 0.442 | 0.178 |
| rs2736340 | T | Intergenic | *BLK* | 0.239 | 0.684 |
| rs26232 | T | Intronic | *C5orf30* | 0.332 | 0.274 |
| rs13315591 | C | Intronic | *FAM107A* | 0.093 | 0.000 |
| rs706778 | T | Intronic | *IL2RA* | 0.403 | 0.542 |

For SNP rs6920220 and rs10499194 located on *TNFAIP3-OLIG3* locus, we assigned *TNFAIP3* because fine-mapping studies of this region identified several SNPs located on coding and intron regions of *TNFAIP3*. For SNP s2736340 located at the intergenic region between *FAM167A* and *BLK*, we assigned BLK for rs2736340 because several GWASs of RA and systemic lupus erythematosus identified SNPs that are located at the intron of BLK and in LD with rs2736340. We could not definitively assign SNPs rs874040 and rs11646922 to a single protein-coding gene. Details are in Text S1C.
