# Supplementary material for: A Systems Genetics Approach Provides a Bridge from Discovered Genetic Variants to Biological Pathways in Rheumatoid Arthritis
Source: PLoS One. 2011 Sep 28;6(9):e25389. doi: 10.1371/journal.pone.0025389 (PMC3182219; doi:10.1371/journal.pone.0025389)
Supplement: Table S6 — Ethnic group-specific analysis of published meta-analyses of genetic associations with RA risk. The SNPs in which the heterogeneity in the ORs between European and East Asian populations are significant are highlighted in yellow. (DOC) [file pone.0025389.s010.doc]

**Table S6A.** Ethnic group (European)-specific analysis of published meta-analyses of genetic associations with RA risk. The SNPs in which the heterogeneity in the ORs between European and East Asian populations are significant are highlighted in yellow.

| European |  |  |  |  |  |  |  |  |  |  |  |  |  |
| --- | --- | --- | --- | --- | --- | --- | --- | --- | --- | --- | --- | --- | --- |
|  |  |  | Sample size | |  |  | Fixed effects model | | |  | Random effects model | | |
| Gene | Polymorphism | Studies | Cases | Controls | *I*^2^ (%) | *P_hetero_* | OR | 95% CI | *P* |  | OR | 95% CI | *P* |
| *STAT4* | rs7574865 | 12 | 13,585 | 15,680 | 60.9 | 0.003 | 1.217 | 1.170-1.265 | 5.6×10^-23^ |  | 1.253 | 1.173-1.339 | 2.4×10^-11^ |
| *FCRL3* | rs7528684 | 9 | 5,645 | 5,592 | 22.8 | 0.240 | 1.031 | 0.977-1.089 | 0.27 |  | 1.038 | 0.974-1.107 | 0.25 |
| *TNF-α* | rs1800629 | 9 | 975 | 1,575 | 71.0 | 5.5×10^-4^ | 0.809 | 0.685-0.955 | 0.012 |  | 0.91 | 0.656-1.262 | 0.57 |
| *TRAF1-C5* | rs3761847 | 11 | 9,592 | 11,563 | 81.9 | 2.9×10^-8^ | 1.161 | 1.116-1.207 | 8.7×10^-14^ |  | 1.244 | 1.123-1.377 | 2.7×10^-5^ |
| *CCL21* | rs2812378 | 12 | 11,011 | 21,194 | 0.0 | 0.60 | 1.109 | 1.069-1.150 | 2.7×10^-8^ |  | 1.109 | 1.069-1.150 | 2.7×10^-8^ |
| *CD40* | rs4810485 | 12 | 10,988 | 21,292 | 7.5 | 0.37 | 0.868 | 0.833-0.904 | 9.8×10^-12^ |  | 0.868 | 0.831-0.907 | 2.6×10^-10^ |
| *CDK6* | rs42041 | 12 | 11,023 | 21,207 | 34.9 | 0.111 | 1.082 | 1.040-1.125 | 9.6×10^-5^ |  | 1.081 | 1.025-1.141 | 4.2×10^-3^ |
| *PADI4* | rs2240340 | 8 | 8,864 | 16,260 | 49.1 | 0.056 | 1.030 | 0.989-1.073 | 0.16 |  | 1.057 | 0.985-1.133 | 0.12 |
| *PTPN22* | rs2476601 | 11 | 6,652 | 7,579 | 18.9 | 0.26 | 1.646 | 1.529-1.771 | 2.0×10^-40^ |  | 1.661 | 1.527-1.806 | 2.2×10^-32^ |
| *SLC22A4* | rs2073838 | 7 | 9,168 | 7,465 | 15.6 | 0.31 | 1.063 | 0.977-1.155 | 0.15 |  | 1.051 | 0.955-1.157 | 0.31 |
| *IL1B* | rs16944 | 7 | 2,852 | 2,319 | 0.0 | 0.69 | 1.107 | 1.019-1.202 | 0.016 |  | 1.107 | 1.019-1.202 | 0.016 |
| *IRF5* | rs2004640 | 8 | 4,624 | 3,742 | 29.9 | 0.19 | 0.881 | 0.828-0.937 | 6.3×10^-5^ |  | 0.880 | 0.814-0.951 | 1.3×10^-3^ |
| *FCGR3A* | rs396991 | 5 | 1,639 | 1,594 | 59.7 | 0.042 | 1.134 | 1.021-1.259 | 0.019 |  | 1.101 | 0.918-1.319 | 0.30 |
| *CTLA4* | rs3087243 | 6 | 4,505 | 4,639 | 18.1 | 0.30 | 0.907 | 0.853-0.964 | 1.8×10^-3^ |  | 0.908 | 0.848-0.972 | 5.8×10^-3^ |
| *TNFAIP3-OLIG3* | rs6920220 | 7 | 8,637 | 9,888 | 0.0 | 0.81 | 1.262 | 1.201-1.326 | 3.9×10^-20^ |  | 1.262 | 1.201-1.326 | 3.9×10^-20^ |
| *CCR5* | delta32 (rs333) | 6 | 2,626 | 3,371 | 43.8 | 0.11 | 0.737 | 0.644-0.845 | 1.1×10^-5^ |  | 0.717 | 0.592-0.869 | 6.7×10^-4^ |
| *TNFRSF1B* | rs1061622 | 6 | 3,393 | 2,071 | 51.0 | 0.070 | 0.985 | 0.898-1.081 | 0.75 |  | 1.030 | 0.885-1.200 | 0.70 |
| *BANK1* | rs17266594 | 3 | 2,988 | 3,400 | 0.0 | 0.64 | 0.998 | 0.922-1.080 | 0.95 |  | 0.998 | 0.922-1.080 | 0.95 |
| *TNFAIP3-OLIG3* | rs10499194 | 5 | 4,533 | 7,399 | 67.5 | 0.015 | 0.820 | 0.771-0.871 | 1.8×10^-10^ |  | 0.802 | 0.716-0.898 | 1.3×10^-4^ |

**Table S6B.** Ethnic group (East Asian)-specific analysis of published meta-analyses of genetic associations with RA risk. The SNPs in which the heterogeneity in the ORs between European and East Asian populations are significant are highlighted in yellow.

| East Asian |  |  |  |  |  |  |  |  |  |  |  |  |  |
| --- | --- | --- | --- | --- | --- | --- | --- | --- | --- | --- | --- | --- | --- |
|  |  |  | Sample size | |  |  | Fixed effects model | | |  | Random effects model | | |
| Gene | Polymorphism | Studies | Cases | Controls | *I*^2^ (%) | *P_hetero_* | OR | 95% CI | *P* |  | OR | 95% CI | *P* |
| *STAT4* | rs7574865 | 4 | 4,563 | 3,091 | 0.0 | 0.73 | 1.267 | 1.183-1.358 | 1.4×10^-11^ |  | 1.267 | 1.183-1.358 | 1.4×10^-11^ |
| *FCRL3* | rs7528684 | 5 | 4,111 | 3,420 | 52.9 | 0.075 | 1.164 | 1.089-1.243 | 7.8×10^-6^ |  | 1.164 | 1.056-1.283 | 2.1×10^-3^ |
| *PADI4* | rs2240340 | 4 | 3,713 | 2,485 | 31.5 | 0.22 | 1.310 | 1.217-1.410 | 5.6×10^-13^ |  | 1.316 | 1.203-1.440 | 2.0×10^-9^ |
| *SLC22A4* | rs2073838 | 4 | 3,549 | 3,034 | 0.0 | 0.50 | 1.157 | 1.074-1.245 | 1.1×10^-4^ |  | 1.157 | 1.074-1.245 | 1.1×10^-4^ |

The SNPs in which the heterogeneity in the ORs between European and East Asian populations are significant are highlighted in yellow.
