# Supplementary material for: A Systems Genetics Approach Provides a Bridge from Discovered Genetic Variants to Biological Pathways in Rheumatoid Arthritis
Source: PLoS One. 2011 Sep 28;6(9):e25389. doi: 10.1371/journal.pone.0025389 (PMC3182219; doi:10.1371/journal.pone.0025389)
Supplement: Table S7 — Genotype counts for six HLA-DRB1 alleles and 15 SNPs. (DOC) [file pone.0025389.s011.doc]

**Table S7.** Genotype counts for six *HLA-DRB1* alleles and 15 SNPs.

| Gene | Locus | SNP | A1/A2^A^ | Cases |  |  |  | Controls |  |  |
| --- | --- | --- | --- | --- | --- | --- | --- | --- | --- | --- |
|  |  |  |  | 1/1 | 1/2 | 2/2 |  | 1/1 | 1/2 | 2/2 |
| *HLA-DRB1* | 6p21.3 | **01:01* | +/- | 7 | 160 | 1,119 |  | 1 | 157 | 1,331 |
|  |  | **09:01* | +/- | 55 | 330 | 901 |  | 24 | 387 | 1,078 |
|  |  | **10:01* | +/- | 0 | 27 | 1,259 |  | 0 | 11 | 1,478 |
|  |  | **04:01* | +/- | 1 | 53 | 1,232 |  | 0 | 34 | 1,455 |
|  |  | **04:04* | +/- | 0 | 9 | 1,277 |  | 0 | 7 | 1,482 |
|  |  | **04:05* | +/- | 76 | 540 | 670 |  | 34 | 349 | 1,106 |
| **SNPs with strong evidence of association (*P*< 2.5×10^-3^)** | | | | | | | | | | |
| *CCR6* | 6q27 | rs3093024 | A/G | 342 | 638 | 301 |  | 313 | 747 | 430 |
| *PADI4* | 1p36.13 | rs2240340 | T/C | 250 | 645 | 389 |  | 235 | 712 | 547 |
| *BLK* | 8p23.1 | rs2736340 | T/C | 639 | 534 | 93 |  | 672 | 648 | 165 |
| *CD40* | 20q13.12 | rs4810485 | T/G | 157 | 550 | 569 |  | 246 | 675 | 569 |
| **SNPs with nominally significant association signals (*P*<0.05)** | | | | | | | | | | |
| *C5orf30* | 5q21.1 | rs26232 | T/C | 78 | 503 | 699 |  | 116 | 621 | 757 |
| *SLC22A4* | 5q31.1 | rs2073838 | A/G | 169 | 553 | 559 |  | 151 | 647 | 692 |
| *AFF3* | 2q11.2 | rs11676922 | T/A | 357 | 627 | 293 |  | 371 | 742 | 378 |
| *FCRL3* | 1q23.1 | rs7528684 | G/A | 236 | 619 | 429 |  | 253 | 682 | 557 |
| **SNPs showing the same direction of effect** | | | | | | | | | | |
| *SPRED2* | 2p14 | rs934734 | G/A | 45 | 392 | 843 |  | 30 | 446 | 1,017 |
| *STAT4* | 2q32.3 | rs7574865 | T/G | 176 | 567 | 537 |  | 169 | 669 | 655 |
| *CTLA4* | 2q33.2 | rs3087243 | A/G | 89 | 457 | 734 |  | 96 | 592 | 798 |
| *TRAF1* | 9q33.2 | rs3761847 | A/G | 290 | 659 | 334 |  | 310 | 780 | 398 |
| *IL2RA* | 10p15.1 | rs706778 | T/C | 414 | 621 | 246 |  | 458 | 738 | 299 |
| **SNPs showing the opposite direction of effect** | | | | | | | | | | |
| *TNFAIP3* | 6q23.3 | rs10499194 | T/C | 8 | 171 | 1,103 |  | 6 | 174 | 1,315 |
| **SNPs excluded from analysis (HWE test *P*<0.001)** | | | | | | | | | | |
| *IRF5* | 7q32.1 | rs2004640 | G/T | 546 | 401 | 287 |  | 676 | 443 | 312 |

^A^ A1 and A2 represent the coded and non-coded alleles, respectively.
