# Supplementary material for: A Systems Genetics Approach Provides a Bridge from Discovered Genetic Variants to Biological Pathways in Rheumatoid Arthritis
Source: PLoS One. 2011 Sep 28;6(9):e25389. doi: 10.1371/journal.pone.0025389 (PMC3182219; doi:10.1371/journal.pone.0025389)
Supplement: Table S8 — Association analysis of RF and anti-CCP positive RA patients versus control subjects with selected genetic variants. (DOC) [file pone.0025389.s012.doc]

**Table S8.** Association analysis of RF and anti-CCP positive RA patients versus control subjects with selected genetic variants.

| Gene | Locus | SNP | A1/A2 | Cases |  |  |  | Controls |  |  |  | Univariate^A^ |  |  | Multivariate^A^ |  |
| --- | --- | --- | --- | --- | --- | --- | --- | --- | --- | --- | --- | --- | --- | --- | --- | --- |
|  |  |  |  | 1/1 | 1/2 | 2/2 |  | 1/1 | 1/2 | 2/2 |  | OR (95% CI) | *P* |  | OR (95% CI) | *P* |
| *HLA-DRB1* | 6p21.3 | **01:01* | +/- | 1 | 43 | 300 |  | 1 | 157 | 1,331 |  | 1.25 (0.88-1.78) | 0.21 |  | 1.78 (1.21-2.61) | 3.3×10^-3^ |
|  |  | **09:01* | +/- | 15 | 93 | 236 |  | 24 | 387 | 1,078 |  | 1.28 (1.02-1.60) | 0.031 |  | 1.97 (1.53-2.54) | 1.2×10^-7^ |
|  |  | **10:01* | +/- | 0 | 10 | 334 |  | 0 | 11 | 1,478 |  | 4.02 (1.69-9.55) | 1.6×10^-3^ |  | 5.12 (2.04-12.8) | 5.0×10^-4^ |
|  |  | **04:01* | +/- | 0 | 18 | 326 |  | 0 | 34 | 1,455 |  | 2.36 (1.32-4.24) | 3.9×10^-3^ |  | 3.35 (1.78-6.30) | 1.8×10^-4^ |
|  |  | **04:04* | +/- | 0 | 3 | 341 |  | 0 | 7 | 1,482 |  | 1.86 (0.48-7.24) | 0.37 |  | 3.39 (0.78-14.6) | 0.10 |
|  |  | **04:05* | +/- | 23 | 152 | 169 |  | 34 | 349 | 1,106 |  | 2.49 (2.04-3.04) | 6.1×10^-19^ |  | 3.08 (2.46-3.86) | 1.5×10^-22^ |
| *CCR6* | 6q27 | rs3093024 | A/G | 103 | 163 | 78 |  | 313 | 747 | 430 |  | 1.35 (1.15-1.60) | 3.7×10^-4^ |  | 1.35 (1.13-1.62) | 8.7×10^-4^ |
| *PADI4* | 1p36.13 | rs2240340 | T/C | 65 | 174 | 105 |  | 235 | 712 | 547 |  | 1.21 (1.02-1.43) | 0.026 |  | 1.24 (1.03-1.49) | 0.020 |
| *BLK* | 8p23.1 | rs2736340 | T/C | 164 | 154 | 26 |  | 672 | 648 | 165 |  | 1.15 (0.96-1.38) | 0.13 |  | 1.16 (0.95-1.40) | 0.15 |
| *CD40* | 20q13.12 | rs4810485 | T/G | 33 | 161 | 149 |  | 246 | 675 | 569 |  | 0.77 (0.65-0.92) | 3.7×10^-3^ |  | 0.77 (0.64-0.93) | 6.3×10^-3^ |
| *C5orf30* | 5q21.1 | rs26232 | T/C | 15 | 141 | 186 |  | 116 | 621 | 757 |  | 0.83 (0.68-1.01) | 0.058 |  | 0.83 (0.67-1.02) | 0.069 |
| *SLC22A4* | 5q31.1 | rs2073838 | A/G | 44 | 134 | 166 |  | 151 | 647 | 692 |  | 1.02 (0.85-1.21) | 0.83 |  | 1.05 (0.86-1.27) | 0.64 |
| *AFF3* | 2q11.2 | rs11676922 | T/A | 92 | 170 | 81 |  | 371 | 742 | 378 |  | 1.08 (0.91-1.27) | 0.39 |  | 1.05 (0.88-1.25) | 0.61 |
| *FCRL3* | 1q23.1 | rs7528684 | G/A | 65 | 156 | 122 |  | 253 | 682 | 557 |  | 1.08 (0.91-1.27) | 0.38 |  | 1.09 (0.92-1.31) | 0.31 |
| *SPRED2* | 2p14 | rs934734 | G/A | 10 | 99 | 234 |  | 30 | 446 | 1,017 |  | 1.03 (0.82-1.29) | 0.80 |  | 1.10 (0.86-1.40) | 0.45 |
| *STAT4* | 2q32.3 | rs7574865 | T/G | 48 | 157 | 138 |  | 169 | 669 | 655 |  | 1.15 (0.97-1.36) | 0.12 |  | 1.14 (0.94-1.37) | 0.17 |
| *CTLA4* | 2q33.2 | rs3087243 | A/G | 19 | 108 | 216 |  | 96 | 592 | 798 |  | 0.75 (0.61-0.92) | 5.6×10^-3^ |  | 0.82 (0.66-1.02) | 0.075 |
| *TRAF1* | 9q33.2 | rs3761847 | A/G | 75 | 175 | 94 |  | 310 | 780 | 398 |  | 1.01 (0.85-1.20) | 0.93 |  | 0.97 (0.81-1.17) | 0.76 |
| *IL2RA* | 10p15.1 | rs706778 | T/C | 123 | 162 | 58 |  | 458 | 738 | 299 |  | 1.18 (1.00-1.40) | 0.049 |  | 1.17 (0.97-1.40) | 0.094 |
| *TNFAIP3* | 6q23.3 | rs10499194 | T/C | 2 | 48 | 292 |  | 6 | 174 | 1,315 |  | 1.24 (0.90-1.71) | 0.19 |  | 1.24 (0.88-1.75) | 0.21 |

^A^ ORs and 95% CIs were estimated by logistic regression analyses using univariate analysis for each allele and then using multivariate analysis including all the allele.
