# Supplementary material for: A Systems Genetics Approach Provides a Bridge from Discovered Genetic Variants to Biological Pathways in Rheumatoid Arthritis
Source: PLoS One. 2011 Sep 28;6(9):e25389. doi: 10.1371/journal.pone.0025389 (PMC3182219; doi:10.1371/journal.pone.0025389)
Supplement: Table S9 — GO and KEGG annotations for three clusters in RA-associated network comprising RA-associated genes and genes ranked in the top 50 by the RWR algorithm. (DOC) [file pone.0025389.s013.doc]

**Table S9.** GO and KEGG annotations for three clusters in RA-associated network comprising RA-associated genes and genes ranked in the top 50 by the RWR algorithm. The same terms as shown in Table 3 were analyzed.

| Annotation | Term | Count^A^ | %^B^ | FE^C^ | P-value |
| --- | --- | --- | --- | --- | --- |
| **Cluster 1** | | | | | |
| GO:0045321 | Leukocyte activation | 14 | 48.3 | 19.1 | 2.8×10^-14^ |
| GO:0002521 | Leukocyte differentiation | 10 | 34.5 | 23.8 | 1.0×10^-10^ |
| hsa04660 | T cell receptor signaling pathway | 13 | 44.8 | 16.2 | 1.5×10^-12^ |
| GO:0006468 | Protein amino acid phosphorylation | 5 | 17.2 | 8.7 | 2.1×10^-3^ |
| **Cluster 2** | | | | | |
| hsa04620 | Toll-like receptor signaling pathway | 7 | 50.0 | 24.3 | 5.3×10^-8^ |
| hsa04622 | RIG-I-like receptor signaling pathway | 5 | 35.7 | 29.5 | 9.8×10^-6^ |
| GO:0007249 | I-kappaB kinase/NF-kappaB cascade | 4 | 28.6 | 36.9 | 1.2×10^-4^ |
| hsa05200 | Pathways in cancer | 4 | 28.6 | 4.1 | 0.051 |
| hsa04623 | Cytosolic DNA-sensing pathway | 3 | 21.4 | 25.2 | 4.9×10^-3^ |
| hsa04621 | NOD-like receptor signaling pathway | 2 | 14.3 | 10.5 | 0.16 |
| **Cluster 3** | | | | | |
| GO:0006935 | Chemotaxis | 9 | 64.3 | 41.1 | 2.5×10-^12^ |
| GO:0007626 | Locomotory behavior | 9 | 64.3 | 24.2 | 1.8×10^-10^ |
| GO:0006955 | Immune response | 9 | 64.3 | 10.7 | 1.2×10^-7^ |
| GO:0006952 | Defense response | 8 | 57.1 | 10.5 | 1.4×10^-6^ |
| GO:0019957 | C-C chemokine binding | 4 | 28.6 | 195.3 | 6.1×10^-7^ |
| GO:0016493 | C-C chemokine receptor activity | 4 | 28.6 | 195.3 | 6.1×10^-7^ |

^A^ Number of GO or KEGG category genes in each cluster.

^B^ Percentage of GO or KEGG category genes in each cluster.

^C^ Fold Enrichment of genes in each cluster compared to a background list.
