# Supplementary material for: A Systems Genetics Approach Provides a Bridge from Discovered Genetic Variants to Biological Pathways in Rheumatoid Arthritis
Source: PLoS One. 2011 Sep 28;6(9):e25389. doi: 10.1371/journal.pone.0025389 (PMC3182219; doi:10.1371/journal.pone.0025389)
Supplement: Table S10 — GO and KEGG annotations for three clusters in RA-associated network comprising RA-associated genes and genes ranked in the top 150 by the RWR algorithm. (DOC) [file pone.0025389.s014.doc]

**Table S10.** GO and KEGG annotations for three clusters in RA-associated network comprising RA-associated genes and genes ranked in the top 150 by the RWR algorithm. The same terms as shown in Table 3 were analyzed.

| Annotation | Term | Count^A^ | %^B^ | FE^C^ | P-value |
| --- | --- | --- | --- | --- | --- |
| **Cluster 1** | | | | | |
| GO:0045321 | Leukocyte activation | 22 | 32.8 | 13.1 | 2.3×10^-18^ |
| GO:0002521 | Leukocyte differentiation | 16 | 23.9 | 16.7 | 1.2×10^-14^ |
| hsa04660 | T cell receptor signaling pathway | 16 | 23.9 | 8.5 | 1.3×10^-10^ |
| GO:0006468 | Protein amino acid phosphorylation | 24 | 35.8 | 5.3 | 1.7×10^-11^ |
| **Cluster 2** | | | | | |
| hsa04620 | Toll-like receptor signaling pathway | 21 | 28.0 | 12.1 | 3.0×10^-17^ |
| hsa04622 | RIG-I-like receptor signaling pathway | 16 | 21.3 | 15.7 | 9.5×10^-15^ |
| GO:0007249 | I-kappaB kinase/NF-kappaB cascade | 13 | 17.3 | 22.7 | 1.9×10^-13^ |
| hsa05200 | Pathways in cancer | 31 | 41.3 | 5.3 | 9.6×10^-16^ |
| hsa04623 | Cytosolic DNA-sensing pathway | 11 | 14.7 | 15.4 | 7.4×10^-10^ |
| hsa04621 | NOD-like receptor signaling pathway | 14 | 18.7 | 12.2 | 2.9×10^-11^ |
| **Cluster 3** | | | | | |
| GO:0006935 | Chemotaxis | 10 | 62.5 | 37.1 | 3.8×10^-13^ |
| GO:0007626 | Locomotory behavior | 10 | 62.5 | 21.9 | 4.7×10^-11^ |
| GO:0006955 | Immune response | 12 | 75.0 | 11.6 | 8.4×10^-11^ |
| GO:0006952 | Defense response | 11 | 68.8 | 11.7 | 1.0×10^-9^ |
| GO:0019957 | C-C chemokine binding | 4 | 25.0 | 165.3 | 1.1×10^-6^ |
| GO:0016493 | C-C chemokine receptor activity | 4 | 25.0 | 165.3 | 1.1×10^-6^ |
| **Cluster 4** | | | | | |
| GO:0005882 | Intermediate filament | 4 | 18.2 | 27.5 | 3.1×10^-4^ |

^A^ Number of GO or KEGG category genes in each cluster.

^B^ Percentage of GO or KEGG category genes in each cluster.

^C^ Fold Enrichment of genes in each cluster compared to a background list.
