# Supplementary material for: A Systems Genetics Approach Provides a Bridge from Discovered Genetic Variants to Biological Pathways in Rheumatoid Arthritis
Source: PLoS One. 2011 Sep 28;6(9):e25389. doi: 10.1371/journal.pone.0025389 (PMC3182219; doi:10.1371/journal.pone.0025389)
Supplement: Table S11 — Re-consideration on RA-associated network. The RWR algorithm was re-examined by adding recently discovered 4 genes (AIRE, CD247, UBASH3A, and ATXN2). GO and KEGG annotations for three clusters in RA-associated network comprising RA-associated genes and genes ranked in the top 100 by the RWR algorithm. (DOC) [file pone.0025389.s015.doc]

**Table S11.** Re-consideration on RA-associated network. The RWR algorithm was re-examined by adding recently discovered 4 genes (*AIRE*, *CD247*, *UBASH3A*, and *ATXN2*). GO and KEGG annotations for three clusters in RA-associated network comprising RA-associated genes and genes ranked in the top 100 by the RWR algorithm. The same terms as shown in Table 3 were analyzed.

| Annotation | Term | Count^A^ | %^B^ | FE^C^ | P-value |
| --- | --- | --- | --- | --- | --- |
| **Cluster 1** | | | | | |
| GO:0045321 | Leukocyte activation | 21 | 41.2 | 16.4 | 8.0×10^-20^ |
| GO:0002521 | Leukocyte differentiation | 16 | 31.4 | 21.8 | 1.2×10^-16^ |
| hsa04660 | T cell receptor signaling pathway | 15 | 29.4 | 10.6 | 2.2×10^-11^ |
| GO:0006468 | Protein amino acid phosphorylation | 21 | 41.2 | 6.0 | 2.1×10^-11^ |
| **Cluster 2** | | | | | |
| hsa04620 | Toll-like receptor signaling pathway | 19 | 34.5 | 16.5 | 2.2×10^-18^ |
| hsa04622 | RIG-I-like receptor signaling pathway | 13 | 23.6 | 19.2 | 5.2×10^-13^ |
| GO:0007249 | I-kappaB kinase/NF-kappaB cascade | 11 | 20.0 | 26.8 | 4.4×10^-12^ |
| hsa05200 | Pathways in cancer | 20 | 36.4 | 5.2 | 5.6×10^-10^ |
| hsa04623 | Cytosolic DNA-sensing pathway | 9 | 16.4 | 18.9 | 9.6×10^-9^ |
| hsa04621 | NOD-like receptor signaling pathway | 10 | 18.2 | 13.1 | 2.8×10^-8^ |
| **Cluster 3** | | | | | |
| GO:0006935 | Chemotaxis | 9 | 56.3 | 33.4 | 3.1×10-^11^ |
| GO:0007626 | Locomotory behavior | 9 | 56.3 | 19.7 | 2.1×10^-9^ |
| GO:0006955 | Immune response | 11 | 68.8 | 10.6 | 2.7×10^-9^ |
| GO:0006952 | Defense response | 10 | 62.5 | 10.7 | 2.8×10^-8^ |
| GO:0019957 | C-C chemokine binding | 4 | 25.0 | 165.3 | 1.1×10^-6^ |
| GO:0016493 | C-C chemokine receptor activity | 4 | 25.0 | 165.3 | 1.1×10^-6^ |

^A^ Number of GO or KEGG category genes in each cluster.

^B^ Percentage of GO or KEGG category genes in each cluster.

^C^ Fold Enrichment of genes in each cluster compared to a background list.
