# Supplementary material for: A Systems Genetics Approach Provides a Bridge from Discovered Genetic Variants to Biological Pathways in Rheumatoid Arthritis
Source: PLoS One. 2011 Sep 28;6(9):e25389. doi: 10.1371/journal.pone.0025389 (PMC3182219; doi:10.1371/journal.pone.0025389)
Supplement: Text S1 — Supplementary methods. (DOC) [file pone.0025389.s016.doc]

**Text S1**

**A. Database search strategies and re-analysis of published meta-analyses**

The 86 articles ^[1-86]^ retrieved by the PubMed search and one relevant article ^[87]^ by the reference mining were numbered serially from 1 to 87. The reference number of this note is seen as ‘Ref ID’ in Tables S1, S2, S3, and S4.

Through our search strategies (Tables S1-S3, Figure S1A), we found 29 articles addressing the associations of 27 polymorphisms located on 18 genetic loci ^[3, 6, 12, 14, 27-29, 35, 36, 43-46, 48, 50-52, 55, 61-64, 66, 68-70, 76, 77, 82]^. When there were several meta-analyses addressing the same polymorphism, we selected the most recent one. When there was a discrepancy in included studies between several meta-analyses on the same polymorphism published around the same time, we judged in each case which studies should be combined. After reducing redundant polymorphisms on the same genetic locus, 20 polymorphisms were re-analyzed in the same statistical manner (detail of statistics is presented in Tables S4 and S6). Among them, the association of 14 polymorphisms passed the statistical threshold at *P*=0.05/20=2.5×10^-3^ by fixed effects model (FEM) meta-analyses: *STAT4* (rs7574865); *CTLA4* (rs3087243); *FCRL3* (rs7528684); *TRAF1-C5* (rs3761847); *CCL21* (rs2812378); *CD40* (rs4810485); *CDK6* (rs42041); *PADI4* (rs2240340); *PTPN22* (rs2476601); *SLC22A4* (rs2073838); *IRF5* (rs2004640); *TNFAIP3-OLIG3* (rs6920220 and rs10499194); and *CCR5* (rs333). Out of the 14 polymorphisms, meta-analyses of 6 polymorphisms showed statistically significant between-study heterogeneity (*P_hetero_*<0.10): *STAT4*; *FCRL3*; *TRAF1-C5*; *PADI4*; *TNFAIP3-OLIG3* (rs10499194); and *CCR5*. Even when applying random effects model (REM) meta-analysis, which is a conservative approach under the presence of between-study heterogeneity, evidence of associations of all the 14 polymorphisms was significant (*P*<0.05).

***STAT4*:** Five articles performed meta-analysis of SNP rs7574865 at the *STAT4* locus on risk of RA ^[12, 36, 51, 62, 76]^. The meta-analysis by Suarez-Gestal et al. ^[76]^ was the most comprehensive one but did not include a replication study performed in Daha et al. ^[12]^ that was published in the same year. Accordingly, a total of 17 studies were included in our re-analysis.

The OR was 1.23 (95% CI: 1.19-1.27, *P*=4.0×10^-34^) and 1.25 (95% CI: 1.19-1.32, *P*=1.7×10^-19^) by FEM and REM meta-analyses, respectively. Between-study heterogeneity was statistically significant (*P_hetero_*=0.013, *I*^2^=48.7%). Regarding the observed heterogeneity, Ji et al. performed a deeper analysis: after removing one study showing outstanding OR and deviation from HWE in controls, the between-study heterogeneity became insignificant ^[36]^.

***CTLA4*:** Three articles investigating two SNPs (rs3087243 and rs231775) at the *CTLA4* locus ^[12, 27, 52]^. These SNPs are in linkage disequilibrium (LD) (*r*^2^=0.48 in CEU samples from HapMap phase III), suggesting that the two SNPs may capture the same association signal. We focused on rs3087243 because of the larger sample size. The meta-analysis on rs3087243 included 8 studies encompassing a total of 11,659 samples ^[12]^. Meta-analysis on rs231775 included 14 studies, but the total of number of samples was 5,748 ^[52]^. Furthermore, the largest GWA meta-analysis supports evidence of association of rs3087243 at the significance level of *P* < 5.0x10^-8^ ^[75]^.

The OR was 0.89 (95% CI: 0.85-0.95, *P*=8.5×10^-5^) under FEM. Because the between-study heterogeneity was moderate (*I*^2^=29.0%, *P_hetero_*=0.20), a similar result was observed under REM (OR=0.89, 95% CI: 0.83-0.95, *P*=9.7×10^-4^).

***FCRL3*:** Four publications on rs7528684 at the *FCRL3* locus were retrieved ^[3, 50, 77, 80]^. Two articles provided the largest list of studies ^[3, 50]^. One article that focused on East Asian populations added one study into the list ^[77]^. Collectively, a total of 14 studies were combined. By FEM meta-analysis, the OR was 1.08 (95% CI: 1.04-1.13, *P*=2.2×10^-4^). The significant evidence of between-study heterogeneity was observed (*P_hetero_*=0.015, *I*^2^=50.8%). By REM meta-analysis, the OR was 1.09 (95% CI: 1.02-1.16, *P*=6.1×10^-3^). Subgroup analysis according to ethnicity identified an association in East Asian populations (4,111 cases and 3,420 controls, OR=1.16, 95% CI: 1.09-1.24, *P*=7.8×10^-6^) but not in European white populations (5,645 cases and 5,592 controls, OR=1.03, 95% CI: 0.98-1.09, *P*=0.27) under FEM. By REM, a similar result was obtained for East Asian populations (OR=1.16, 95% CI: 1.06-1.28, *P*=2.1×10^-3^) and for European white populations (OR=1.04, 95% CI: 0.97-1.11, *P*=0.25). The recent European GWA meta-analysis captured a weak association signal of this SNP (OR=1.08, *P*=0.001) ^[75]^.

***TRAF1-C5*:** Patsopoulos et al. reported meta-analyses of two SNPs (rs3761847 and rs2900180) at the *TRAF1*-*C5* region ^[64]^. These two SNPs are in LD (*r*^2^=0.62). We chose rs3761847 in terms of the number of studies and sample size. The OR with FEM was highly significant (OR=1.13, 95% CI: 1.09-1.17, *P*=9.2×10^-11^). The between-study heterogeneity was very large (*I*^2^=83.4%, *P_hetero_*=1.1×10^-10^). Therefore, the confidence interval of the OR with REM was much wider and the statistical significance weakened (OR=1.18, 95% CI: 1.07-1.31, *P*=8.1×10^-4^).

***CCL21*:** Two articles investigated the association of rs2812378 at the *CCL21* locus ^[63, 70]^. The meta-analysis of three GWA datasets identified the association of *CCL21*, *CD40* and *CDK6* ^[70]^. Orozco et al. then performed a large-scale replication study and combined their replication cohort with the original GWA meta-analysis.^[63]^ The significance of rs2812378 in the replication cohort was modest (OR=1.08, 95% CI: 1.01-1.16, *P*=0.04). There is no evidence of between-study heterogeneity (*I*^2^=0.0%, *P_hetero_*=0.60). Both under FEM and REM, consistent evidence of association was observed (OR=1.11, 95% CI: 1.07-1.15, *P*=2.7×10^-8^).

***CD40*:** As described in *CCL21*, the association between rs4810485 at the *CD40* locus was examined in two articles ^[63, 70]^. The association of rs4810485 in the replication cohort was strong (OR=0.87, 95% CI: 0.80-0.94, *P*=3.6×10^-4^). The between-study heterogeneity was small (*I*^2^=0.0%, *P_hetero_*=0.60). The OR was 0.87 (95% CI: 0.83-0.90, *P*=9.8×10^-12^) and 0.87 (95% CI: 0.83-0.91, *P*=2.6×10^-10^) by FEM and REM meta-analyses, respectively.

***CDK6*:** Two articles performed meta-analyses of rs42041 at the *CDK6* locus ^[63, 70]^. The association of rs42041 failed to replicate in the large UK cohort (OR=1.00, 95% CI: 0.92-1.08, *P*=0.91). The meta-analysis shows that the association signal became weaker by combining the replication cohort with the original GWA meta-analysis (OR=1.08, 95% CI: 1.04-1.13, *P*=9.6×10^-5^ under FEM and OR=0.87, 95% CI: 0.80-0.94, *P*=4.2×10^-3^ under REM). The between-study heterogeneity was moderate (*I*^2^=34.9%, *P_hetero_*=0.11).

***PADI4*:** Five articles examined meta-analyses of rs2240340 at the *PADI4* locus ^[6, 35, 46, 68, 77]^. Although Lee et al. ^[46]^ analyzed the other two SNPs (rs1748033 and rs874881), we re-analyzed rs2240340, which had been the most comprehensively analyzed. Among these articles, two and one articles focused on European white ^[6, 68]^ and East Asian ^[77]^ populations, respectively. Combining the two ethnic group-specific meta-analyses provided the most comprehensive list of studies ^[6, 77]^. Collectively, a total of 12 studies were synthesized. Although the between-study heterogeneity was very large (*I*^2^=77.8%, *P_hetero_*=7.6×10^-7^), the association was significant in the overall populations both under FEM (OR=1.09, 95% CI: 1.05-1.13, *P*=2.0×10^-6^) and under REM (OR=1.16, 95% CI: 1.06-1.27, *P*=7.0×10^-4^). As previously described ^[35, 46, 88]^, the magnitude of association was stronger in East Asian than in European populations (*P_hetero-groups_*=2.1×10^-8^). The OR in East Asian was highly significant (OR=1.31, 95% CI: 1.22-1.41, *P*=5.6×10^-13^) under FEM and (OR=1.32, 95% CI: 1.20-1.44, *P*=2.0×10^-9^) under REM. 3,713 cases and 2,485 controls were analyzed in East Asian subgroup.

***PTPN22*:** Lee et al. analyzed the contribution of rs2476601 at the *PTPN22* locus ^[48]^. Consistent evidence of association was observed (*I*^2^=16.7%, *P_hetero_*=0.28). The OR was 1.64 (95% CI: 1.52-1.76, *P*=2.0×10^-40^) and 1.65 (95% CI: 1.52-1.79, *P*=7.3×10^-33^) by FEM and REM, respectively.

***SLC22A4*:** Two articles addressing the association between rs2073838 at the SLC22A4 locus have been published ^[61, 66]^. The association was significant in the overall populations both by FEM (OR=1.11, 95% CI: 1.05-1.18, *P*=1.3×10^-4^) and by REM (OR=1.11, 95% CI: 1.04-1.18, *P*=9.7×10^-4^). The between-study heterogeneity was low (*I*^2^=14.3%, *P_hetero_*=0.31), and the difference in the summary OR between East Asian and European populations was not significant (*P_hetero-groups_*=0.14).

***IRF5*:** We retrieved two articles analyzing 4 SNPs (rs2004640, rs2280714, rs729302 and rs752637) at the *IRF5* locus ^[14, 28]^. We selected rs2004640 because the SNP was most comprehensively analyzed ^[28]^. By FEM, the OR was 0.90 (95% CI: 0.86-0.95, *P*=1.4×10^-4^). Since the between-study heterogeneity was moderate (*I*^2^=30.3%, *P_hetero_*=0.17), a similar result was observed by REM (OR=0.90, 95% CI: 0.84-0.96, *P*=1.7×10^-3^). In the former meta-analysis, the effect of this SNP was stronger in anti-CCP negative patients than in anti-CCP positive patients ^[14]^. This intriguing finding should be corroborated by further replication studies.

***TNFAIP3-OLIG3*:** Patsopoulos et al. performed meta-analyses of two SNPs (rs6920220 and rs10499194) at the *TNFAIP3-OLIG3* locus ^[64]^. These two SNPs are in weak LD (*r*^2^=0.04) and therefore are likely to be independently associated with risk of RA.

For rs6920220, results from 7 studies were combined. Consistent evidence of association was observed (*I*^2^=0.0%, *P_hetero_*=0.81). By both FEM and REM, the OR was 1.26 (95% CI: 1.20-1.33, *P*=3.9×10^-20^).

For rs10499194, results from five studies were combined. There was large heterogeneity among studies (*I*^2^=67.5%, *P_hetero_*=0.015). The OR was highly significant under FEM (OR=0.82, 95% CI: 0.77-0.87, *P*=1.8×10^-10^), whereas the confidence interval became much wider and the significance weakened under REM (OR=0.80, 95% CI: 0.72-0.90, *P*=1.3×10^-4^).

***CCR5*:** Three articles performed meta-analyses of rs333, which is a 32 base-pair deletion (*CCR5-Δ32*), at the *CCR5* locus ^[55, 69, 82]^. Combining the studies included in Wheeler et al. ^[82]^ with those in Lindner et al. ^[55]^ provided a comprehensive list of studies addressing the association between rs333 and RA. Collectively, the results from seven studies were combined in our re-analysis. One study from the Latin American population showed opposite direction, but the sample size was low (142 cases and 70 controls) and the allele frequency of rs333 was rare (0% and 2.1% in controls and cases, respectively). In overall populations, the OR under FEM was 0.74 (95% CI: 0.65-0.85, *P*=1.5×10^-5^). Although there was moderate between-study heterogeneity observed (*I*^2^=45.9%, *P_hetero_*=0.085), the association was significant by REM (OR=0.72, 95% CI: 0.59-0.89, *P*=1.8×10^-3^).

*T****NF-α*:** The association between rs1800629 and RA was assessed in one meta-analysis ^[45]^. Results from individual studies were extremely inconsistent (*P_hetero_*=2.6×10^-9^, *I*^2^=81.6%). In overall populations, there was no evidence of association. The result implies different associations according to ethnicity. However, the limited sample size in each ethnic group (Latin American, 390 cases and 634 controls; East Asian, 97 cases and 97 controls). A larger sample size may be needed for reliable subgroup analysis.

***IL1B***: For the *IL1B* locus, two SNPs (rs16944 and rs1143634) were analyzed by two articles ^[29, 43]^. These two SNPs are in weak LD (*r*^2^=0.05). For rs16944, Lee et al.^[43]^ was the latest report but missed three studies that were included in Harrison et al. that focused on European descent populations ^[29]^. Collectively, we synthesized 11 studies. In overall populations, there was no evidence of association with RA. In European descent populations (2,852 cases and 2,319 controls), the association was modestly significant (OR=1.11, 95% CI: 1.02-1.20, *P*=0.016).

The association of rs1143634 was modestly significant in overall populations by FEM (OR=0.83, 95% CI: 0.73-0.95, *P*=6.0×10^-3^). However, the association diminished by REM (OR=0.80, 95% CI: 0.63-1.02, *P*=0.066) due to large between-study heterogeneity (*P_hetero_*=2.4×10^-3^, *I*^2^=64.8%). In subgroup analysis of East Asian populations, the association was significant. However, the sample size was quite low in the East Asian subgroup (519 cases and 534 controls). Further large-scale replication studies should be accumulated to corroborate this subgroup-specific association.

***FCGR3A*:** Lee et al. performed meta-analyses of rs396991, rs1801274 and NA1-NA2 isoforms at the *FCGR3A*, *FCGR2A* and *FCGR3B* loci, respectively ^[44]^. We exclude *FCGR2A* and *FCGR3B* because the sample sizes were less than 3,000. Results from nine studies were synthesized. There was no evidence of association for rs396991 at *FCGR3A*.

***TNFRSF1B*:** Plenge et al. performed replication study and meta-analysis of SNP rs1061622 at the *TNFRSF1B* locus ^[68]^. The number of studies included was six. The association was not statistically significant.

***BANK1*:** Suarez-Gestal et al. examined association of SNP rs17266594 at the *BANK1* locus ^[76]^. Five studies were combined. The association was not statistically significant.

**B. The setting of ‘base model’ in the simulation study**

We set a base model where all the possible combinations of genotypes of the *HLA-DRB1* alleles and the selected SNPs were included. For the *HLA-DRB1* locus, the combination of the six putative risk alleles (*HLA-DRB1*01:01*, *DRB1*09:01*, *DRB1*10:01*, *DRB1*04:04*, *DRB1*04:01*, and *DRB1*04:05*) and one referent group comprising the other alleles generated 28 genotypes. For the SNPs, the calculation of three to the 14th power resulted in 4,782,969 genotypes. Collectively, the base model generated 133,923,132 multi-locus genotypes were generated. To simulate the distribution of RA risks in the general population of Japanese, the ORs and the allele frequencies of 14 SNPs and *HLA-DRB1* alleles are needed. The ORs and the allele frequencies for *HLA-DRB1* alleles are as follows:

| *HLA-DRB1* allele | OR | Allele frequency |
| --- | --- | --- |
| *DRB1*01:01* | 1.78 | 0.0571 |
| *DRB1*09:01* | 1.97 | 0.1452 |
| *DRB1*10:01* | 5.12 | 0.0048 |
| *DRB1*04:04* | 3.39 | 0.0020 |
| *DRB1*04:01* | 3.35 | 0.0102 |
| *DRB1*04:05* | 3.08 | 0.1327 |
| Others (referent) | 1.00 | 0.6480 |

The ORs were obtained with the multivariate logistic regression analysis for RF and anti-CCP positive patients (Table S7). The allele frequencies were retrieved from Central Bone Marrow Data Center (<http://www.bmdc.jrc.or.jp/stat.html>).

The ORs and the allele frequencies for 14 SNPs were as follows:

| SNP ID | Risk allele | OR | Allele frequency |
| --- | --- | --- | --- |
| rs7574865 (*STAT4*) | G | 1.2 | 0.355 |
| rs7528684 (*FCRL3*) | T | 1.2 | 0.427 |
| rs3761847 (*TRAF1*) | A | 1.1 | 0.451 |
| rs4810485 (*CD40*) | G | 1.2 | 0.594 |
| rs2240340 (*PADI4*) | T | 1.3 | 0.390 |
| rs2073838 (*SLC22A4*) | A | 1.1 | 0.326 |
| rs3087243 (*CTLA4*) | G | 1.1 | 0.742 |
| rs10499194 (*TNFAIP3*) | T | 1.2 | 0.909 |
| rs3093024 (*CCR6*) | A | 1.2 | 0.431 |
| rs11676922 (*AFF3*) | T | 1.2 | 0.500 |
| rs934734 (*SPRED2*) | G | 1.1 | 0.178 |
| rs2736340 (*BLK*) | C | 1.2 | 0.684 |
| rs26232 (*C5orf30*) | C | 1.1 | 0.726 |
| rs706778 (*IL2RA*) | T | 1.1 | 0.542 |

The ORs were obtained with the reanalysis of published meta-analyses (Table 1). The allele frequencies were obtained from the SNP Control Database ^[89]^. The ORs and the frequencies for risk alleles are shown.

Using these ORs and the allele frequencies, the probability of multi-locus genotypes given disease status was calculated by the equation [3] in the main text. The genetic profiles of multi-locus genotypes were sorted in descending order of their risk. By sliding risk threshold, the ROC curve was drawn by plotting the TPR versus the FPR (expressed as the equation [4] in the main text). The figure below is the ROC curve for the base model. The resulting AUC was 0.71.


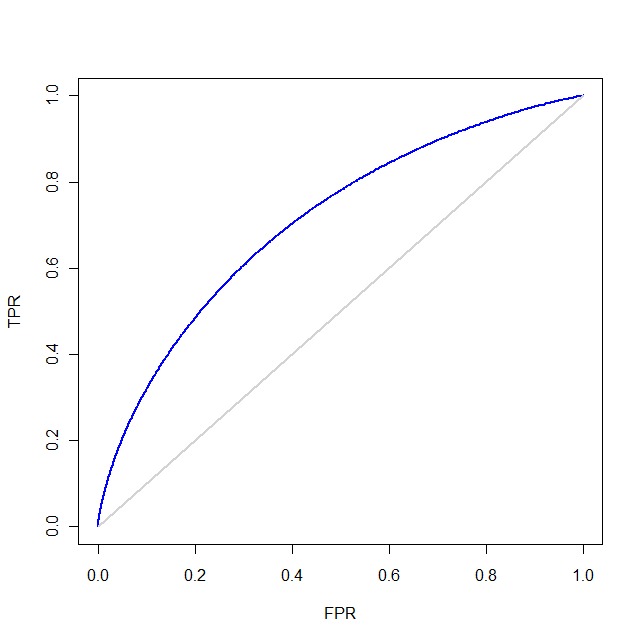


**C. Assignment of selected variants to a single protein-coding gene for the network analysis**

We assigned selected genetic variants to a single protein-coding gene according to the following hierarchy: coding > intronic > 5’UTR > 3’UTR > near gene (within 2kb to 5’ or 0.5kb to 3’ of a gene) > intergenic. The result is shown in Table S5.

**Variants in coding region:** The SNP rs2476601 is a non-synonymous change substituting arginine for tryptophan at amino acid residue 620 of *PTPN22*. Rs333 is a 32bp deletion polymorphism and causes a frameshift at amino acid 185 of *CCR5*.

**Variants in intron region:** Among the selected 24 variants, 13 SNPs are in the intron regions (*STAT4*, *CD40*, *CDK6*, *PADI4*, *SLC22A4*, *IRF5*, *CCR6*, *REL*, *ANKRD55*, *SPRED2*, *C5orf30*, *FAM107A* and *IL2RA*).

**Variants in UTR region.** The rs3761847 is located on 5’ UTR of *TRAF1*.

**Variants within 2kb to 5’ or within 0.5 kb to 3’ of a gene:** Rs7528684 and rs2812378 are at 5’ upstream of *FCRL3* and *CCL21*, respectively. The SNP 6230G>A also known as CT60 (rs3087243) is localized at 3’ of the known polyadenilation site of *CTLA4*. The distances from these three SNPs to the assigned genes are very close (~1kb).

**Variants at integenic region:** The five SNPs (rs6920220, rs10499194, rs2736340, rs11676922 and rs874040) map intergenic regions. Rs6920220 and rs10499194 are localized at *TNFAIP3-OLIG3* region. We assigned *TNFAIP3* because fine-mapping studies of this region identified several SNPs located on coding and intron regions of *TNFAIP3* ^[90, 91]^. Rs2736340 is located at the intergenic region between *FAM167A* and *BLK.* We assigned *BLK* for rs2736340 because several GWASs of RA and systemic lupus erythematosus identified SNPs that are located at the intron of *BLK* and in LD with rs2736340 ^[92-95]^. Rs11676922 was located at the intergeneic region between *AFF3* and *LONRF2*. Rs11676922 are 80kb apart from 5’ of *AFF3*. This SNP is 8kb distant from 3’ of *LONRF2*. The association of SNPs of *AFF3-LONRF2* region has been consistent ^[92, 96]^. Rs874040 is localized at the intergenic region between *SEL1L3* and *RBPJ*. Rs874040 is 21kb apart from 5’ region of *RBPJ* and 24kb apart from 5’ of *SLE1L3*. We could not definitively assign these two SNPs to a single protein-coding gene.

References

1.     Alizadeh BZ, Valdigem G, Coenen MJ, Zhernakova A, Franke B, et al. (2007) Association analysis of functional variants of the FcgRIIa and FcgRIIIa genes with type 1 diabetes, celiac disease and rheumatoid arthritis. Hum Mol Genet 16(21): 2552-2559. 10.1093/hmg/ddm194.

2.     Barnetche T, Constantin A, Cantagrel A, Cambon-Thomsen A, Gourraud PA. (2008) New classification of HLA-DRB1 alleles in rheumatoid arthritis susceptibility: A combined analysis of worldwide samples. Arthritis Res Ther 10(1): R26. 10.1186/ar2379.

3.     Begovich AB, Chang M, Schrodi SJ. (2007) Meta-analysis evidence of a differential risk of the FCRL3 -169T-->C polymorphism in white and east asian rheumatoid arthritis patients. Arthritis Rheum 56(9): 3168-3171. 10.1002/art.22857.

4.     Briollais L, Durrieu G, Upathilake R. (2007) Novel approach for genome scan meta-analysis of rheumatoid arthritis: A kernel-based estimation procedure. BMC Proc 1 Suppl 1: S96.

5.     Bronson PG, Criswell LA, Barcellos LF. (2008) The MHC2TA -168A/G polymorphism and risk for rheumatoid arthritis: A meta-analysis of 6861 patients and 9270 controls reveals no evidence for association. Ann Rheum Dis 67(7): 933-936. 10.1136/ard.2007.077099.

6.     Burr ML, Naseem H, Hinks A, Eyre S, Gibbons LJ, et al. (2010) PADI4 genotype is not associated with rheumatoid arthritis in a large UK caucasian population. Ann Rheum Dis 69(4): 666-670. 10.1136/ard.2009.111294.

7.     Carr EJ, Niederer HA, Williams J, Harper L, Watts RA, et al. (2009) Confirmation of the genetic association of CTLA4 and PTPN22 with ANCA-associated vasculitis. BMC Med Genet 10: 121. 10.1186/1471-2350-10-121.

8.     Chang WW, Su H, He L, Zhao KF, Wu JL, et al. (2010) Association between transforming growth factor-beta1 T869C polymorphism and rheumatoid arthritis: A meta-analysis. Rheumatology (Oxford) 49(4): 652-656. 10.1093/rheumatology/kep417.

9.     Chelala C, Duchatelet S, Joffret ML, Bergholdt R, Dubois-Laforgue D, et al. (2007) PTPN22 R620W functional variant in type 1 diabetes and autoimmunity related traits. Diabetes 56(2): 522-526. 10.2337/db06-0942.

10.     Choi SJ, Rho YH, Ji JD, Song GG, Lee YH. (2006) Genome scan meta-analysis of rheumatoid arthritis. Rheumatology (Oxford) 45(2): 166-170. 10.1093/rheumatology/kei128.

11.     Coenen MJ, Trynka G, Heskamp S, Franke B, van Diemen CC, et al. (2009) Common and different genetic background for rheumatoid arthritis and coeliac disease. Hum Mol Genet 18(21): 4195-4203. 10.1093/hmg/ddp365.

12.     Daha NA, Kurreeman FA, Marques RB, Stoeken-Rijsbergen G, Verduijn W, et al. (2009) Confirmation of STAT4, IL2/IL21, and CTLA4 polymorphisms in rheumatoid arthritis. Arthritis Rheum 60(5): 1255-1260. 10.1002/art.24503.

13.     Delgado-Vega AM, Anaya JM. (2007) Meta-analysis of HLA-DRB1 polymorphism in latin american patients with rheumatoid arthritis. Autoimmun Rev 6(6): 402-408. 10.1016/j.autrev.2006.11.004.

14.     Dieguez-Gonzalez R, Calaza M, Perez-Pampin E, de la Serna AR, Fernandez-Gutierrez B, et al. (2008) Association of interferon regulatory factor 5 haplotypes, similar to that found in systemic lupus erythematosus, in a large subgroup of patients with rheumatoid arthritis. Arthritis Rheum 58(5): 1264-1274. 10.1002/art.23426.

15.     Etzel CJ, Chen WV, Shepard N, Jawaheer D, Cornelis F, et al. (2006) Genome-wide meta-analysis for rheumatoid arthritis. Hum Genet 119(6): 634-641. 10.1007/s00439-006-0171-8.

16.     Eyre S, Flynn E, Martin P, Hinks A, Wilson AG, et al. (2010) No evidence for association of the KLF12 gene with rheumatoid arthritis in a large UK cohort. Ann Rheum Dis 69(7): 1407-1408. 10.1136/ard.2009.120428.

17.     Eyre S, Hinks A, Flynn E, Martin P, Wilson AG, et al. (2010) Confirmation of association of the REL locus with rheumatoid arthritis susceptibility in the UK population. Ann Rheum Dis 69(8): 1572-1573. 10.1136/ard.2009.122887.

18.     Festen EA, Goyette P, Scott R, Annese V, Zhernakova A, et al. (2009) Genetic variants in the region harbouring IL2/IL21 associated with ulcerative colitis. Gut 58(6): 799-804. 10.1136/gut.2008.166918.

19.     Fisher MC, Cronstein BN. (2009) Metaanalysis of methylenetetrahydrofolate reductase (MTHFR) polymorphisms affecting methotrexate toxicity. J Rheumatol 36(3): 539-545. 10.3899/jrheum.080576.

20.     Fisher SA, Lanchbury JS, Lewis CM. (2003) Meta-analysis of four rheumatoid arthritis genome-wide linkage studies: Confirmation of a susceptibility locus on chromosome 16. Arthritis Rheum 48(5): 1200-1206. 10.1002/art.10945.

21.     Glinskii AB, Ma J, Ma S, Grant D, Lim CU, et al. (2009) Identification of intergenic trans-regulatory RNAs containing a disease-linked SNP sequence and targeting cell cycle progression/differentiation pathways in multiple common human disorders. Cell Cycle 8(23): 3925-3942.

22.     Gorman JD, Criswell LA. (2002) The shared epitope and severity of rheumatoid arthritis. Rheum Dis Clin North Am 28(1): 59-78.

23.     Gorman JD, David-Vaudey E, Pai M, Lum RF, Criswell LA. (2004) Particular HLA-DRB1 shared epitope genotypes are strongly associated with rheumatoid vasculitis. Arthritis Rheum 50(11): 3476-3484. 10.1002/art.20588.

24.     Gorman JD, David-Vaudey E, Pai M, Lum RF, Criswell LA. (2004) Lack of association of the HLA-DRB1 shared epitope with rheumatoid nodules: An individual patient data meta-analysis of 3,272 caucasian patients with rheumatoid arthritis. Arthritis Rheum 50(3): 753-762. 10.1002/art.20119.

25.     Gorman JD, Lum RF, Chen JJ, Suarez-Almazor ME, Thomson G, et al. (2004) Impact of shared epitope genotype and ethnicity on erosive disease: A meta-analysis of 3,240 rheumatoid arthritis patients. Arthritis Rheum 50(2): 400-412. 10.1002/art.20006.

26.     Graham RR, Cotsapas C, Davies L, Hackett R, Lessard CJ, et al. (2008) Genetic variants near TNFAIP3 on 6q23 are associated with systemic lupus erythematosus. Nat Genet 40(9): 1059-1061. 10.1038/ng.200.

27.     Han S, Li Y, Mao Y, Xie Y. (2005) Meta-analysis of the association of CTLA-4 exon-1 +49A/G polymorphism with rheumatoid arthritis. Hum Genet 118(1): 123-132. 10.1007/s00439-005-0033-9.

28.     Han SW, Lee WK, Kwon KT, Lee BK, Nam EJ, et al. (2009) Association of polymorphisms in interferon regulatory factor 5 gene with rheumatoid arthritis: A metaanalysis. J Rheumatol 36(4): 693-697. 10.3899/jrheum.081054.

29.     Harrison P, Pointon JJ, Chapman K, Roddam A, Wordsworth BP. (2008) Interleukin-1 promoter region polymorphism role in rheumatoid arthritis: A meta-analysis of IL-1B-511A/G variant reveals association with rheumatoid arthritis. Rheumatology (Oxford) 47(12): 1768-1770. 10.1093/rheumatology/ken374.

30.     Hinks A, Ke X, Barton A, Eyre S, Bowes J, et al. (2009) Association of the IL2RA/CD25 gene with juvenile idiopathic arthritis. Arthritis Rheum 60(1): 251-257. 10.1002/art.24187.

31.     Hollis-Moffatt JE, Merriman ME, Rodger RA, Rowley KA, Chapman PT, et al. (2009) Evidence for association of an interleukin 23 receptor variant independent of the R381Q variant with rheumatoid arthritis. Ann Rheum Dis 68(8): 1340-1344. 10.1136/ard.2008.090142.

32.     Hollis-Moffatt JE, Rowley KA, Phipps-Green AJ, Merriman ME, Dalbeth N, et al. (2009) The ITGAV rs3738919 variant and susceptibility to rheumatoid arthritis in four caucasian sample sets. Arthritis Res Ther 11(5): R152. 10.1186/ar2828.

33.     Ioannidis JP, Tarassi K, Papadopoulos IA, Voulgari PV, Boki KA, et al. (2002) Shared epitopes and rheumatoid arthritis: Disease associations in greece and meta-analysis of mediterranean european populations. Semin Arthritis Rheum 31(6): 361-370.

34.     Issekutz A, Belagyi T, Racz I, Olah A. (2003) Membranous constriction of the colon caused by non-steroidal anti-inflammatory drugs--case report]. Magy Seb 56(3-4): 120-122.

35.     Iwamoto T, Ikari K, Nakamura T, Kuwahara M, Toyama Y, et al. (2006) Association between PADI4 and rheumatoid arthritis: A meta-analysis. Rheumatology (Oxford) 45(7): 804-807. 10.1093/rheumatology/kel023.

36.     Ji JD, Lee WJ, Kong KA, Woo JH, Choi SJ, et al. (2010) Association of STAT4 polymorphism with rheumatoid arthritis and systemic lupus erythematosus: A meta-analysis. Mol Biol Rep 37(1): 141-147. 10.1007/s11033-009-9553-z.

37.     Jun KR, Choi SE, Cha CH, Oh HB, Heo YS, et al. (2007) Meta-analysis of the association between HLA-DRB1 allele and rheumatoid arthritis susceptibility in asian populations. J Korean Med Sci 22(6): 973-980.

38.     Katchamart W, Johnson S, Lin HJ, Phumethum V, Salliot C, et al. (2010) Predictors for remission in rheumatoid arthritis patients: A systematic review. Arthritis Care Res (Hoboken) 62(8): 1128-1143. 10.1002/acr.20188.

39.     Kim YJ, Park JH, Kim I, Kim JO, Bae JS, et al. (2008) Putative role of functional interferon regulatory factor 5 (IRF5) polymorphism in rheumatoid arthritis in a korean population. J Rheumatol 35(11): 2106-2118.

40.     Kurreeman FA, Goulielmos GN, Alizadeh BZ, Rueda B, Houwing-Duistermaat J, et al. (2010) The TRAF1-C5 region on chromosome 9q33 is associated with multiple autoimmune diseases. Ann Rheum Dis 69(4): 696-699. 10.1136/ard.2008.106567.

41.     Lee YC, Raychaudhuri S, Cui J, De Vivo I, Ding B, et al. (2009) The PRL -1149 G/T polymorphism and rheumatoid arthritis susceptibility. Arthritis Rheum 60(5): 1250-1254. 10.1002/art.24468.

42.     Lee YH, Ji JD, Bae SC, Song GG. (2010) Associations between tumor necrosis factor-alpha (TNF-alpha) -308 and -238 G/A polymorphisms and shared epitope status and responsiveness to TNF-alpha blockers in rheumatoid arthritis: A metaanalysis update. J Rheumatol 37(4): 740-746. 10.3899/jrheum.090707.

43.     Lee YH, Ji JD, Song GG. (2009) Association between interleukin 1 polymorphisms and rheumatoid arthritis susceptibility: A metaanalysis. J Rheumatol 36(1): 12-15. 10.3899/jrheum.080450.

44.     Lee YH, Ji JD, Song GG. (2008) Associations between FCGR3A polymorphisms and susceptibility to rheumatoid arthritis: A metaanalysis. J Rheumatol 35(11): 2129-2135.

45.     Lee YH, Ji JD, Song GG. (2007) Tumor necrosis factor-alpha promoter -308 A/G polymorphism and rheumatoid arthritis susceptibility: A metaanalysis. J Rheumatol 34(1): 43-49.

46.     Lee YH, Rho YH, Choi SJ, Ji JD, Song GG. (2007) PADI4 polymorphisms and rheumatoid arthritis susceptibility: A meta-analysis. Rheumatol Int 27(9): 827-833. 10.1007/s00296-007-0320-y.

47.     Lee YH, Rho YH, Choi SJ, Ji JD, Song GG. (2006) Association of TNF-alpha -308 G/A polymorphism with responsiveness to TNF-alpha-blockers in rheumatoid arthritis: A meta-analysis. Rheumatol Int 27(2): 157-161. 10.1007/s00296-006-0175-7.

48.     Lee YH, Rho YH, Choi SJ, Ji JD, Song GG, et al. (2007) The PTPN22 C1858T functional polymorphism and autoimmune diseases--a meta-analysis. Rheumatology (Oxford) 46(1): 49-56. 10.1093/rheumatology/kel170.

49.     Lee YH, Song GG. (2010) Associations between the C677T and A1298C polymorphisms of MTHFR and the efficacy and toxicity of methotrexate in rheumatoid arthritis: A meta-analysis. Clin Drug Investig 30(2): 101-108. 10.2165/11531070-000000000-00000; 10.2165/11531070-000000000-00000.

50.     Lee YH, Woo JH, Choi SJ, Ji JD, Song GG. (2010) Fc receptor-like 3 -169 C/T polymorphism and RA susceptibility: A meta-analysis. Rheumatol Int 30(7): 947-953. 10.1007/s00296-009-1082-5.

51.     Lee YH, Woo JH, Choi SJ, Ji JD, Song GG. (2010) Association between the rs7574865 polymorphism of STAT4 and rheumatoid arthritis: A meta-analysis. Rheumatol Int 30(5): 661-666. 10.1007/s00296-009-1051-z.

52.     Lei C, Dongqing Z, Yeqing S, Oaks MK, Lishan C, et al. (2005) Association of the CTLA-4 gene with rheumatoid arthritis in chinese han population. Eur J Hum Genet 13(7): 823-828. 10.1038/sj.ejhg.5201423.

53.     Lin DY, Sullivan PF. (2009) Meta-analysis of genome-wide association studies with overlapping subjects. Am J Hum Genet 85(6): 862-872. 10.1016/j.ajhg.2009.11.001.

54.     van der Linden MP, Feitsma AL, le Cessie S, Kern M, Olsson LM, et al. (2009) Association of a single-nucleotide polymorphism in CD40 with the rate of joint destruction in rheumatoid arthritis. Arthritis Rheum 60(8): 2242-2247. 10.1002/art.24721.

55.     Lindner E, Nordang GB, Melum E, Flato B, Selvaag AM, et al. (2007) Lack of association between the chemokine receptor 5 polymorphism CCR5delta32 in rheumatoid arthritis and juvenile idiopathic arthritis. BMC Med Genet 8: 33. 10.1186/1471-2350-8-33.

56.     Maiti AK, Kim-Howard X, Viswanathan P, Guillen L, Rojas-Villarraga A, et al. (2010) Confirmation of an association between rs6822844 at the Il2-Il21 region and multiple autoimmune diseases: Evidence of a general susceptibility locus. Arthritis Rheum 62(2): 323-329. 10.1002/art.27222.

57.     McKinney C, Fanciulli M, Merriman ME, Phipps-Green A, Alizadeh BZ, et al. (2010) Association of variation in fcgamma receptor 3B gene copy number with rheumatoid arthritis in caucasian samples. Ann Rheum Dis 69(9): 1711-1716. 10.1136/ard.2009.123588.

58.     Merriman TR, Cordell HJ, Eaves IA, Danoy PA, Coraddu F, et al. (2001) Suggestive evidence for association of human chromosome 18q12-q21 and its orthologue on rat and mouse chromosome 18 with several autoimmune diseases. Diabetes 50(1): 184-194.

59.     Nishino M, Ikegami H, Fujisawa T, Kawaguchi Y, Kawabata Y, et al. (2005) Functional polymorphism in Z-DNA-forming motif of promoter of SLC11A1 gene and type 1 diabetes in japanese subjects: Association study and meta-analysis. Metabolism 54(5): 628-633. 10.1016/j.metabol.2004.12.006.

60.     O'Rielly DD, Roslin NM, Beyene J, Pope A, Rahman P. (2009) TNF-alpha-308 G/A polymorphism and responsiveness to TNF-alpha blockade therapy in moderate to severe rheumatoid arthritis: A systematic review and meta-analysis. Pharmacogenomics J 9(3): 161-167. 10.1038/tpj.2009.7.

61.     Okada Y, Mori M, Yamada R, Suzuki A, Kobayashi K, et al. (2008) SLC22A4 polymorphism and rheumatoid arthritis susceptibility: A replication study in a japanese population and a metaanalysis. J Rheumatol 35(9): 1723-1728.

62.     Orozco G, Alizadeh BZ, Delgado-Vega AM, Gonzalez-Gay MA, Balsa A, et al. (2008) Association of STAT4 with rheumatoid arthritis: A replication study in three european populations. Arthritis Rheum 58(7): 1974-1980. 10.1002/art.23549.

63.     Orozco G, Eyre S, Hinks A, Ke X, Wellcome Trust Case Control consortium YEAR Consortium, et al. (2010) Association of CD40 with rheumatoid arthritis confirmed in a large UK case-control study. Ann Rheum Dis 69(5): 813-816. 10.1136/ard.2009.109579.

64.     Patsopoulos NA, Ioannidis JP. (2010) Susceptibility variants for rheumatoid arthritis in the TRAF1-C5 and 6q23 loci: A meta-analysis. Ann Rheum Dis 69(3): 561-566. 10.1136/ard.2009.109447.

65.     Pavy S, Toonen EJ, Miceli-Richard C, Barrera P, van Riel PL, et al. (2010) Tumour necrosis factor alpha -308G->A polymorphism is not associated with response to TNFalpha blockers in caucasian patients with rheumatoid arthritis: Systematic review and meta-analysis. Ann Rheum Dis 69(6): 1022-1028. 10.1136/ard.2009.117622.

66.     Plant D, Barton A, Thomson W, Ke X, Eyre S, et al. (2009) A re-evaluation of three putative functional single nucleotide polymorphisms in rheumatoid arthritis. Ann Rheum Dis 68(8): 1373-1375. 10.1136/ard.2008.103572.

67.     Plant D, Flynn E, Mbarek H, Dieude P, Cornelis F, et al. (2010) Investigation of potential non-HLA rheumatoid arthritis susceptibility loci in a european cohort increases the evidence for nine markers. Ann Rheum Dis 69(8): 1548-1553. 10.1136/ard.2009.121020.

68.     Plenge RM, Padyukov L, Remmers EF, Purcell S, Lee AT, et al. (2005) Replication of putative candidate-gene associations with rheumatoid arthritis in >4,000 samples from north america and sweden: Association of susceptibility with PTPN22, CTLA4, and PADI4. Am J Hum Genet 77(6): 1044-1060. 10.1086/498651.

69.     Prahalad S. (2006) Negative association between the chemokine receptor CCR5-Delta32 polymorphism and rheumatoid arthritis: A meta-analysis. Genes Immun 7(3): 264-268. 10.1038/sj.gene.6364298.

70.     Raychaudhuri S, Remmers EF, Lee AT, Hackett R, Guiducci C, et al. (2008) Common variants at CD40 and other loci confer risk of rheumatoid arthritis. Nat Genet 40(10): 1216-1223. 10.1038/ng.233.

71.     Raychaudhuri S, Thomson BP, Remmers EF, Eyre S, Hinks A, et al. (2009) Genetic variants at CD28, PRDM1 and CD2/CD58 are associated with rheumatoid arthritis risk. Nat Genet 41(12): 1313-1318. 10.1038/ng.479.

72.     Segurado R, Hamshere ML, Glaser B, Nikolov I, Moskvina V, et al. (2007) Combining linkage data sets for meta-analysis and mega-analysis: The GAW15 rheumatoid arthritis data set. BMC Proc 1 Suppl 1: S104.

73.     Silva GL, Junta CM, Mello SS, Garcia PS, Rassi DM, et al. (2007) Profiling meta-analysis reveals primarily gene coexpression concordance between systemic lupus erythematosus and rheumatoid arthritis. Ann N Y Acad Sci 1110: 33-46. 10.1196/annals.1423.005.

74.     Somers EC, Thomas SL, Smeeth L, Hall AJ. (2006) Autoimmune diseases co-occurring within individuals and within families: A systematic review. Epidemiology 17(2): 202-217. 10.1097/01.ede.0000193605.93416.df.

75.     Stahl EA, Raychaudhuri S, Remmers EF, Xie G, Eyre S, et al. (2010) Genome-wide association study meta-analysis identifies seven new rheumatoid arthritis risk loci. Nat Genet 42(6): 508-514. 10.1038/ng.582.

76.     Suarez-Gestal M, Calaza M, Dieguez-Gonzalez R, Perez-Pampin E, Pablos JL, et al. (2009) Rheumatoid arthritis does not share most of the newly identified systemic lupus erythematosus genetic factors. Arthritis Rheum 60(9): 2558-2564. 10.1002/art.24748.

77.     Takata Y, Inoue H, Sato A, Tsugawa K, Miyatake K, et al. (2008) Replication of reported genetic associations of PADI4, FCRL3, SLC22A4 and RUNX1 genes with rheumatoid arthritis: Results of an independent japanese population and evidence from meta-analysis of east asian studies. J Hum Genet 53(2): 163-173. 10.1007/s10038-007-0232-4.

78.     Tapper W, Collins A, Morton NE. (2007) Mapping a gene for rheumatoid arthritis on chromosome 18q21. BMC Proc 1 Suppl 1: S18.

79.     Telenti A, Egger M. (2007) Identifying safety concerns from genetic data: Lessons from the development of CCR5 inhibitors. Antivir Ther 12(2): 147-148.

80.     Thabet MM, Wesoly J, Slagboom PE, Toes RE, Huizinga TW. (2007) FCRL3 promoter 169 CC homozygosity is associated with susceptibility to rheumatoid arthritis in dutch caucasians. Ann Rheum Dis 66(6): 803-806. 10.1136/ard.2006.064949.

81.     Thomas DC. (2009) Genome-wide association studies for discrete traits. Genet Epidemiol 33 Suppl 1: S8-12. 10.1002/gepi.20465.

82.     Wheeler J, McHale M, Jackson V, Penny M. (2007) Assessing theoretical risk and benefit suggested by genetic association studies of CCR5: Experience in a drug development programme for maraviroc. Antivir Ther 12(2): 233-245.

83.     Williams RC, Jacobsson LT, Knowler WC, del Puente A, Kostyu D, et al. (1995) Meta-analysis reveals association between most common class II haplotype in full-heritage native americans and rheumatoid arthritis. Hum Immunol 42(1): 90-94.

84.     van der Woude D, Lie BA, Lundstrom E, Balsa A, Feitsma AL, et al. (2010) Protection against anti-citrullinated protein antibody-positive rheumatoid arthritis is predominantly associated with HLA-DRB1*1301: A meta-analysis of HLA-DRB1 associations with anti-citrullinated protein antibody-positive and anti-citrullinated protein antibody-negative rheumatoid arthritis in four european populations. Arthritis Rheum 62(5): 1236-1245. 10.1002/art.27366.

85.     Yu X, Wieczorek S, Franke A, Yin H, Pierer M, et al. (2009) Association of UCP2 -866 G/A polymorphism with chronic inflammatory diseases. Genes Immun 10(6): 601-605. 10.1038/gene.2009.29.

86.     Zintzaras E, Ioannidis JP. (2005) Heterogeneity testing in meta-analysis of genome searches. Genet Epidemiol 28(2): 123-137. 10.1002/gepi.20048.

87.     Fernando MM, Stevens CR, Walsh EC, De Jager PL, Goyette P, et al. (2008) Defining the role of the MHC in autoimmunity: A review and pooled analysis. PLoS Genet 4(4): e1000024. 10.1371/journal.pgen.1000024.

88.     Kochi Y, Suzuki A, Yamada R, Yamamoto K. (2009) Genetics of rheumatoid arthritis: Underlying evidence of ethnic differences. J Autoimmun 32(3-4): 158-162. 10.1016/j.jaut.2009.02.020.

89.     Koike A, Nishida N, Inoue I, Tsuji S, Tokunaga K. (2009) Genome-wide association database developed in the japanese integrated database project. J Hum Genet 54(9): 543-546. 10.1038/jhg.2009.68.

90.     Orozco G, Hinks A, Eyre S, Ke X, Gibbons LJ, et al. (2009) Combined effects of three independent SNPs greatly increase the risk estimate for RA at 6q23. Hum Mol Genet 18(14): 2693-2699. 10.1093/hmg/ddp193.

91.     Shimane K, Kochi Y, Horita T, Ikari K, Amano H, et al. (2010) The association of a nonsynonymous single-nucleotide polymorphism in TNFAIP3 with systemic lupus erythematosus and rheumatoid arthritis in the japanese population. Arthritis Rheum 62(2): 574-579. 10.1002/art.27190.

92.     Freudenberg J, Lee HS, Han BG, Shin HD, Kang YM, et al. (2011) Genome-wide association study of rheumatoid arthritis in koreans: Population-specific loci as well as overlap with european susceptibility loci. Arthritis Rheum 63(4): 884-893. 10.1002/art.30235; 10.1002/art.30235.

93.     Hom G, Graham RR, Modrek B, Taylor KE, Ortmann W, et al. (2008) Association of systemic lupus erythematosus with C8orf13-BLK and ITGAM-ITGAX. N Engl J Med 358(9): 900-909. 10.1056/NEJMoa0707865.

94.     International Consortium for Systemic Lupus Erythematosus Genetics (SLEGEN), Harley JB, Alarcon-Riquelme ME, Criswell LA, Jacob CO, et al. (2008) Genome-wide association scan in women with systemic lupus erythematosus identifies susceptibility variants in ITGAM, PXK, KIAA1542 and other loci. Nat Genet 40(2): 204-210. 10.1038/ng.81.

95.     Graham RR, Cotsapas C, Davies L, Hackett R, Lessard CJ, et al. (2008) Genetic variants near TNFAIP3 on 6q23 are associated with systemic lupus erythematosus. Nat Genet 40(9): 1059-1061. 10.1038/ng.200.

96.     Barton A, Eyre S, Ke X, Hinks A, Bowes J, et al. (2009) Identification of AF4/FMR2 family, member 3 (AFF3) as a novel rheumatoid arthritis susceptibility locus and confirmation of two further pan-autoimmune susceptibility genes. Hum Mol Genet 18(13): 2518-2522. 10.1093/hmg/ddp177.
